# Transcriptional PBR cycles at pericentromeric repeats cause gross chromosomal rearrangements through Rad52-dependent ADR-loop formation

**DOI:** 10.1093/nar/gkaf1455

**Published:** 2026-01-13

**Authors:** Ran Xu, Crystal Tang, Jianfang N Wang, Daisuke Motooka, Hideo Tsubouchi, Hiroshi Iwasaki, Takuro Nakagawa

**Affiliations:** Department of Biological Sciences, Graduate School of Science, The University of Osaka, Toyonaka, Osaka 560-0043, Japan; Forefront Research Center, Graduate School of Science, The University of Osaka, Toyonaka, Osaka 560-0043, Japan; Center for Education in Liberal Arts and Sciences, The University of Osaka, Toyonaka, Osaka 560-0043, Japan; Department of Biological Sciences, Graduate School of Science, The University of Osaka, Toyonaka, Osaka 560-0043, Japan; Department of Biological Sciences, Graduate School of Science, The University of Osaka, Toyonaka, Osaka 560-0043, Japan; Research Institute for Microbial Diseases, The University of Osaka, Suita, Osaka 565-0871, Japan; Cell Biology Center, Institute of Integrated Research, Institute of Science Tokyo, Yokohama, Kanagawa 226-8501, Japan; School of Life Science and Technology, Institute of Science Tokyo, Yokohama, Kanagawa 226-8501, Japan; Cell Biology Center, Institute of Integrated Research, Institute of Science Tokyo, Yokohama, Kanagawa 226-8501, Japan; School of Life Science and Technology, Institute of Science Tokyo, Yokohama, Kanagawa 226-8501, Japan; Department of Biological Sciences, Graduate School of Science, The University of Osaka, Toyonaka, Osaka 560-0043, Japan; Forefront Research Center, Graduate School of Science, The University of Osaka, Toyonaka, Osaka 560-0043, Japan; Center for Education in Liberal Arts and Sciences, The University of Osaka, Toyonaka, Osaka 560-0043, Japan; Institute for Radiation Sciences, The University of Osaka, Toyonaka, Osaka 560-0043, Japan

## Abstract

Heterochromatin marked by histone H3 lysine 9 (H3K9) methylation represses transcription of pericentromeric repeats, thereby suppressing gross chromosomal rearrangements (GCRs). However, it remains unclear how transcription causes GCRs when heterochromatin is lost. Using fission yeast, we show that transcriptional Pausing–Backtracking–Restart (PBR) cycles accumulate R-loops, leading to GCRs. DNA–RNA immunoprecipitation (DRIP) revealed that loss of Clr4, the H3K9 methyltransferase, increased R-loops at pericentromeric repeats. Overexpression of RNaseH1 in *clr4∆* cells reduced both R-loops and GCRs, demonstrating that R-loops cause GCRs. Tfs1/TFIIS and Ubp3, required for transcriptional restart, and Seb1, involved in pausing at pericentromeres, were required for R-loop accumulation and GCRs, implicating PBR cycles in the formation of genotoxic R-loops. We also demonstrate that Rad52 recombinase localizes to pericentromeric repeats and facilitates GCRs in *clr4∆* cells. *rad52–R45K*, which impairs single-strand annealing (SSA), reduced GCRs. A single-stranded DNA (ssDNA) region within an R-loop may anneal to homologous ssDNA to form Annealing-induced DNA–RNA-loops (ADR-loops). Indeed, Rad52 facilitated ADR-loop formation *in vitro*. Polδ was also involved in GCRs. These data suggest that, when heterochromatin is lost, transcriptional PBR cycles accumulate R-loops at pericentromeric repeats, and Rad52-dependent SSA converts R-loops into ADR-loops followed by Polδ-dependent break-induced replication (BIR), resulting in homology-mediated GCRs.

## Graphical Abstract

**Graphical Abstract:**
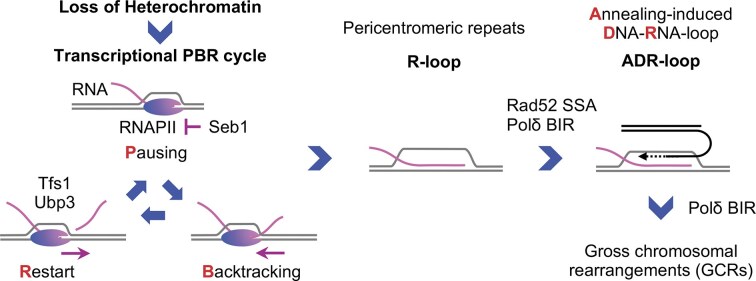

## Introduction

Centromeres are essential chromosomal regions that ensure genome stability through proper chromosomal segregation. Centromeres are characterized by a unique chromatin organization in which the histone H3 variant CENP-A localizes to the central core and promotes kinetochore assembly [1, 2]. The CENP-A chromatin domain is flanked by centromeric and pericentromeric heterochromatin characterized by H3 lysine 9th di- and trimethylation (H3K9me2/3). The heterochromatin surrounding the CENP-A chromatin is involved in various processes, including the proper attachment of microtubules to kinetochores, replication timing, sister centromere cohesion, and transcriptional silencing [3, 4]. Together, these epigenetic features define centromere identity.

Despite their pivotal role in genome maintenance, DNA sequences of centromeres and pericentromeres vary extensively among species and even within the same species [5]. However, centromeres and pericentromeres in many eukaryotes share a common feature: repetitive sequences. For example, in humans, each centromere contains tens of thousands of alpha-satellite (αSat) repeats, flanked by other types of repeats, such as hSat1, 2, or 3. These centromeric and pericentromeric repeats, in total, represent 6.2% of the human genome [6]. CENP-A chromatin is usually formed within the largest higher-order repeat (HOR) array of αSat tandem repeats on each chromosome. Only a small portion, rather than the entire region, of the HOR array is bound by CENP-A [7]. The active HOR domain forming CENP-A chromatin is flanked by heterochromatic HORs and pericentromeres, containing αSat monomers, transposable elements, segmental duplications, and non-αSat repeats either in tandem or inverted orientation. Under normal physiological conditions, the central CENP-A chromatin is transcriptionally active, whereas the flanking centromeric and pericentromeric heterochromatin is transcriptionally silenced. In the fission yeast *Schizosaccharomyces pombe*, the central unique sequence (cnt) is flanked by sets of inverted repeats (imr, dg, dh, and irc). CENP-A chromatin is formed on the cnt unique sequence and the inner part of imr repeats, whereas heterochromatin is assembled on the outer part of imr and other repeats. A recent study showed that repetitive sequences promote heterochromatin formation in fission yeast [8]. However, repetitive sequences also present a risk. Recombination between repetitive sequences can result in homology-mediated gross chromosomal rearrangements (GCRs) [9, 10]. In *S. pombe* and *Candida albicans*, intrachromosomal translocation between centromeric inverted repeats results in the formation of isochromosomes whose arms are mirror images [11–14]. In humans, whole-arm chromosome translocations, including isochromosomes, are observed in various cancers, suggesting a link between centromeric GCRs and tumorigenesis [15–18].

Heterochromatin safeguards centromere integrity via transcriptional silencing [19]. Previously, we showed that loss of Clr4, the H3K9me2/3 methyltransferase, or its regulatory protein Rik1 increased isochromosome formation in fission yeast [20]. Notably, mutating Rpb1, the catalytic subunit of RNA polymerase II (RNAPII), reduced isochromosome formation in *clr4* deletion (*clr4∆*) cells, demonstrating that RNAPII-mediated transcription leads to centromeric GCRs. DNA sequences and DNA-binding proteins can interfere with the elongation of transcription [21]. An RNAPII-binding protein, Seb1, that facilitates heterochromatin formation causes transcriptional pausing at pericentromeric repeats [22–24]. After RNAPII pausing and backtracking on template DNA, RNAPII restarts transcription with the aid of Tfs1/TFIIS, which stimulates Rpb1’s RNA cleavage activity to create a new 3′-end for reinitiating RNA synthesis [25, 26]. Alternatively, RNAPII is degraded in a ubiquitin-dependent manner [27]. Ubp3, a ubiquitin protease, removes ubiquitin from RNAPII to prevent the ubiquitin-dependent degradation, thereby facilitating transcriptional restart [28]. Strikingly, Tfs1 and Ubp3 promote isochromosome formation in *clr4∆* cells [19, 20], implicating transcriptional restart in GCR induction. However, the molecular link between transcription dynamics and GCRs remains poorly defined.

R-loops, three-stranded nucleic acid structures, are formed when nascent RNA hybridizes with template DNA to form DNA–RNA hybrids and evicts the nontemplate DNA strand. R-loops are frequently found at highly transcribed gene bodies, at promoter-proximal pausing sites, and at transcriptional termination sites [29–31]. Depending on the structure and the binding protein, R-loops exhibit different roles. While some R-loops play a role in transcription regulation, others lead to genome instability. In humans, DNA methylation collaborates with H3K9me2/3 modification and forms heterochromatin at pericentromeric repeats [32]. A mutation in the DNA methyltransferase Dnmt3b or related factors results in the Immunodeficiency, Centromere instability, and Facial anomalies (ICF) syndrome, which exhibits chromosome entanglement at pericentromeres [33, 34]. ICF cells exhibit a defect in transcriptional silencing and accumulate R-loops and γH2AX, indicative of DNA damage, at pericentromeric repeats [35, 36]. Overexpression of RNaseH1, which degrades RNA in DNA–RNA hybrids, mitigates R-loops and γH2AX accumulation, suggesting that R-loops cause pericentromere instability in ICF cells. However, the molecular mechanism by which R-loops accumulate and cause pericentromeric GCRs remains unknown.

In this study, we show that loss of heterochromatin leads to R-loop accumulation at pericentromeric repeats via the transcriptional Pausing–Backtracking–Restart (PBR) cycle, resulting in homology-mediated GCRs in fission yeast. DNA–RNA immunoprecipitation (DRIP) revealed the accumulation of R-loops at pericentromeric repeats in *clr4∆* and *rik1∆* cells. Overexpression of RNaseH1 in *clr4∆* cells reduced both R-loops and GCR rates, demonstrating that R-loops cause GCRs. Notably, mutations in *tfs1, ubp3*, or *seb1*, but not a transcription elongation factor, *leo1*, reduced R-loops, showing that PBR cycles accumulate R-loops. The *rad52–R45K* mutation, which specifically impairs single-strand annealing (SSA) [37] but not *rad51∆* or *rad55∆*, reduced GCRs in *clr4∆* cells. Chromatin immunoprecipitation (ChIP) revealed that *clr4∆* increased Rad52, but not Rad51, localization at pericentromeric repeats. RNaseH1 overexpression or the *tfs1* or *seb1* mutation reduced the pericentromeric localization of Rad52, suggesting that the Rad52 localization at pericentromeric repeats depends on R-loop formation. *In vitro*, the Rad52 protein, but not the Rad51 protein, facilitated the formation of Annealing-induced DNA–RNA-loops (ADR-loops) from synthetic R-loops and single-stranded DNA (ssDNA). DNA polymerase delta (Polδ), which is involved in break-induced replication (BIR) and chromosomal DNA replication, was also required for GCRs in *clr4∆* cells. These findings demonstrate a mechanistic pathway in which PBR cycles generate genotoxic R-loops, which, in turn, promote homology-mediated GCRs via Rad52-dependent ADR-loop formation followed by Polδ-dependent BIR.

## Materials and methods

### Yeast media

The fission yeast, *S. pombe*, was grown in yeast extract (YE) medium, Edinburgh minimal medium (EMM), yeast nitrogen base (YNB), 5-fluoroorotic acid (5FOA), or malt extract (ME) medium supplemented with amino acids or bases at a final concentration of 225 mg/l unless otherwise indicated [38]. The yeast cells were grown at 30°C, unless otherwise indicated. YE medium contained 5 g/l YE (Nacalai Tesque, 15838-45) and 30 g/l glucose (Nacalai Tesque, 16805-64). EMM medium contained 5 g/l ammonium chloride (Nacalai Tesque, 02424-55), 3 g/l potassium hydrogen phthalate (Nacalai Tesque, 28420-95), 5.55 g/l di-sodium hydrogen phosphate dodecahydrate (Nacalai Tesque, 31723-35), 20 g/l glucose, 20 ml/l 50× salt stock, 1ml/l 1000× vitamin stock, and 0.1 ml/l 10 000× mineral stock [38]. YNB medium contained 1.8 g/l YNB (BD Difco, 233 520), 5.2 g/l ammonium sulfate (Nacalai Tesque, 02619-15), and 20 g/l glucose. To prepare 5FOA medium, YNB medium is supplemented with 1 g/l 5FOA (Apollo Scientific, PC4054) and 56 mg/l uracil (Tokyo Chemical Industry CO., Ltd., 66-22-8). Solid media, except ME, contained 15 g/l agar (Nacalai Tesque, 01028-85). ME media contained 30 g/l bacteriological MEs (Biokar, A1101HA), adenine (Nacalai Tesque, 06398-82), uracil, and 20 g/l Bacto agar (BD Difco, 214 010).

### Yeast strains and plasmids

The yeast strains and oligonucleotides used in this study are listed in Supplementary Tables S1 and S2, respectively. Yeast strains were created by transformation using the lithium acetate/PEG method [39] or tetrad dissection [38]. The yeast transformation was confirmed by polymerase chain reaction (PCR) analysis. The strains containing the kanamycin, hygromycin, or nourseothricin resistance gene were selected on the medium supplemented with 100 µg/ml of G418 (Nacalai Tesque, 09380-86), hygromycin B (Nacalai Tesque, 09287-84), or 50 µg/ml clonNAT (Werner BioAgents, 5.001.000).

The *Padh1-rnh1* gene was introduced at the Z locus [40] near *spbpb7E8.01* by yeast transformation using pTN1264 plasmid digested with ApaI. pTN1264 was created as follows. A 0.8 kb genomic region containing the *adh1* promoter was amplified using Bgl-Padh1/Padh1-Hind primers, digested with BglII and HindIII, and introduced between BglII–HindIII sites of pFA6a-natMX6 [41] using T4 DNA ligase (Nippon gene, 311-00404), creating pTN1114. A 1.2 kb fragment containing the Z locus was amplified using Zlocus.FOR/Zlocus.REV primers and pNATZA21-cnp3C-CFP-TEV [40], and introduced at the BstXI site of pTN1114 using Gibson assembly master mix (New England Biolabs, E2611S), creating pTN1233. A 0.9 kb fragment containing *rnh1* complementary DNA (cDNA) with 6His3Flag at the C-terminal was amplified using Rnh1.FOR/Rnh1.REV primers and introduced between PvuII–AatII sites of pTN1233 using Gibson assembly master mix, creating pTN1264.

The *rad52-FL, rad52-NM*, and *rad52-N* genes were introduced into the Z locus by yeast transformation using ApaI-digested pTN1275, pTN1277, and pTN1276, respectively. pTN1275, 1277, and 1276 were created as follows. A 1.4 kb MluI-SacI fragment containing the *hphMX6* gene from pFA6a–hphMX6 [41] was introduced between MluI–SacI sites of pTN1264, creating pTN1268. A 2.9 kb region containing the *rad52-6His3Flag* gene was amplified using TNF7567 genomic DNA and rad52-N-F1/kanMX6-UP primers. A 2.1 kb BamHI–SalI restriction fragment of the PCR product was introduced between BglII–SalI sites of pTN1268, creating pTN1275 (Rad52-FL). A 1.4 kb rad52-NM and a 0.3 kb rad52–NLS regions were amplified from pTN1275 using rad22-1/rad52-R_327 and rad52-F_327/oligo211 primer pairs, respectively. To connect them, we performed the second PCR in the presence of the 1.4- and 0.3-kb fragments and rad22-1/oligo211 primers. A 1.2 kb BglII-SalI restriction fragment of the PCR product was introduced between BglII–SalI sites of pTN1275, creating pTN1277 (Rad52-NM). A 1.0 kb rad52-N and a 0.3 kb rad52–NLS regions were amplified from pTN1275 using rad22-1/rad52-R_209 and rad52-F_209/oligo211 primer pairs, respectively. To connect them, we performed the second PCR in the presence of the 1.0- and 0.3-kb fragments and rad22-1/oligo211 primers. A 0.9 kb BglII–SalI restriction fragment of the PCR product was introduced between BglII–SalI sites of pTN1275, creating pTN1276 (Rad52-N). All versions of Rad52 were expressed under the endogenous *rad52* promoter at the Z locus.

The *rad52-6His3Flag* and *rad52-R45K-6His3Flag* genes were expressed in *E. coli* using pTN1287 and pTN1298, respectively. pTN1287 and 1298 were created as follows. The *rad52* gene, codon-optimized for expression in *E. coli*, was cloned into pUC-GW-kan, creating pTN1286 (Genewiz from Azenta Life Sciences). A 1.5 kb NdeI–AvrII fragment containing the *rad52* gene from pTN1286 was introduced between NdeI–AvrII sites of pTN1118 [37], creating pTN1287. To introduce the *rad52-R45K* mutation, we performed the first round of PCR using pTN1286 and rad52p-F1/rad52p-R45K-R and rad52p-R45K-F/rad52p-R1 primer pairs, producing 0.4- and 0.5-kb fragments, respectively. To connect them, we performed the second PCR in the presence of the 0.4- and 0.5-kb fragments and rad52p-F1/rad52p-R1 primers. A 0.6 kb MluI–HindIII restriction fragment of the PCR product was introduced between MluI–HindIII sites of pTN1286, creating pTN1289. A 1.5 kb NdeI–AvrII fragment containing the *rad52-R45K* gene from pTN1289 was introduced between NdeI-AvrII sites of pTN1287, creating pTN1298.

The *tfs1-D274, E275∆* strain was produced by yeast transformation using NdeI-digested pTN1310, which was constructed as follows. A 2.7 kb genomic region containing the *tfs1* gene was amplified using tfs1-1/tfs1-5 primers and digested using EcoRI. The 1.9 kb EcoRI restriction fragment was introduced between EcoRI–BsaAI sites of pTN782 containing *ura4*+ [20], creating pTN1237. A 1.0 kb PCR fragment was prepared using pTN1237 and ura4-chk2_F/tfs1-AcD primers. We performed the second PCR in the presence of the 1.0 kb PCR product, pTN1237, and ura4-chk2_F/tfs1-4 primers. A 1.5 kb NdeI–EcoRI restriction fragment of the PCR product was introduced between NdeI–EcoRI sites of pTN1237, creating pTN1310. After yeast transformation, *ura4*+ clones were selected on EMM plates. Then, the *ura4*+ pop-out clones were selected on 5FOA plates. DNA sequencing confirmed that no additional mutations were introduced into the PCR fragment during plasmid construction.

The *rad52-fmNeonGreen:hphMX6* strain was constructed by PCR-based gene targeting using pTN1252, which was created as follows. The *fmNeonGreen* gene, which encodes the monomeric NeonGreen codon-optimized to be expressed in fission yeast, was cloned into pUC57, creating pTN1250 (Genewiz from Azenta Life Sciences). A 0.7 kb AscI–BamHI fragment containing the *fmNeonGreen* gene from pTN1250 was introduced between AscI–BamHI sites of pFA6a-GFP(S65T)-hphMX6 [42], creating pTN1252.

### DNA–RNA immunoprecipitation assay

7 × 10^8^ yeast cells were harvested from log-phase YE cultures, suspended in TE 10:25 [10 mM Tris–HCl, pH 8.0, 25 mM ethylenediaminetetraacetic acid (EDTA)], and stored overnight at 4°C. To examine the effect of a transcription inhibitor, we added a 100 mg/ml stock solution of 1,10-phenanthroline (Nacalai Tesque, 26708) in ethanol to log-phase EMM cultures to a final concentration of 200 µg/ml. As a mock control, we added an equal volume of ethanol to cultures. The cultures were further incubated for 3 h before harvesting cells.

Cells were resuspended in 1 ml of SP1 buffer (20 mM sodium citrate, 20 mM Na_2_HPO_4_, 40 mM EDTA, pH 5.6). After adding 10 µl of β-mercaptoethanol (Nacalai Tesque, 21418-55), the cell suspension was incubated at 30°C for 20 min with rotation. After centrifugation at 3200 × *g* at 25°C for 1 min using a swing rotor TMS-21, cells were resuspended in 500 µl of SP1 buffer. After adding 50 µl of 3.5 mg/ml lyticase (Sigma–Aldrich, L4025), the cell suspension was incubated at 37°C for ≤50 min until ~30% of the cells became spheroplasts. After centrifugation at 800 × *g* at 25°C for 2 min using the swing rotor, spheroplasts were suspended in 300 µl of TE 50:20 (50 mM Tris–HCl, pH 8.0, 20 mM EDTA). After adding 100 µl of 10% sodium dodecyl sulfate (SDS) (Nacalai Tesque, 31607-65), the suspension was incubated at 50°C for 1 h. After adding 300 µl of 5 M potassium acetate (Nacalai Tesque, 28405-05), the tube was kept on ice for 10 min. After centrifugation at 19 900 × *g* at 4°C for 5 min using the swing rotor, the supernatant was transferred to a new tube containing 750 µl of isopropanol and kept on ice for 10 min. After centrifugation at 17 900 × *g* at 4°C for 5 min using a TOMY micro centrifuge Kitman with an angle rotor, the pellet was rinsed with 1 ml of 70% ethanol and resuspended in 300 µl TE 10:1 (10 mM Tris–HCl, pH 8.0, 1 mM EDTA). Nucleic acid fragmentation was performed using a Sonifier 250 (Branson) at setting 2.5 for 10 s, repeated 4 times with 3-min intervals on ice, and confirmed by gel electrophoresis. After two rounds of phenol/chloroform extraction (Nacalai Tesque, 25967-74), 200 µl of the aqueous phase was recovered, mixed with 10 µl of 5 M NaCl (Nacalai Tesque, 31320-76), 4 µl of glycogen (Nacalai Tesque, 17110-11), and 600 µl of ethanol, and kept at −80°C for 1 h. After centrifugation at 17 900 × *g* at 4°C for 15 min using an angle rotor, the pellet was rinsed with 500 µl of 70% ethanol and resuspend in 100 µl of TE 10:1. The concentration of nucleic acids was determined by NanoDrop One (Thermo Fisher Scientific) and adjusted to 800 ng/µl using TE 10:1. To prepare the input sample, the nucleic acid solution was diluted 100-fold with elution buffer (10 mM Tris–HCl, pH 8.0, 1 mM EDTA, 1% SDS). Forty microliters of the nucleic acid solution (800 ng/µl) was transferred to each of two low protein-binding tubes (BIO-BIK, SC-0150). Fifty microliters of H_2_O and 10 µl of 10× RNaseH reaction buffer were added to each tube. After adding 0.5 µl of 60 U/µl RNaseH (Takara, 2150A) to one of the two tubes, the tubes were incubated at 37°C for 2 h.

In a low protein-binding tube, 20 µl of Dynabeads Protein G (Invitrogen, 10004D) was incubated at 4°C overnight with 2 µl of 1 mg/ml S9.6 antibody (Kerafast, ENH001) in 400 µl of 1× PBS (137 mM NaCl, 27 mM KCl, 10 mM Na_2_HPO_4_, 1.8 mM KH_2_PO_4_, pH 7.4) supplemented with 2% bovine serum albumin (Sigma–Aldrich, A7906). The beads were washed with 400 µl of 1% Lysis buffer (100 mM HEPES-KOH, pH 7.4, 140 mM NaCl, 1 mM EDTA, 1% Triton X-100, 0.1% Na-deoxycholate) and suspended with 310 µl of 1% Lysis buffer. After adding 90 µl of the nucleic acid solution incubated in the presence or absence of RNaseH, the bead suspension was incubated at 4°C for 2 h with rotation. Beads were washed twice with 400 µl of 1% Lysis buffer, once with 400 µl of 1% Lysis buffer supplemented with 500 mM NaCl, twice with 400 µl of Wash buffer (10 mM Tris–HCl, pH 8.0, 1 mM EDTA, pH 8.0, 250 mM LiCl, 0.5% NP40, 0.5% Na-deoxycholate), and once with 400 µl of TE 10:1. To release DNA–RNA hybrids, beads were suspended in 60 µl of elution buffer and incubated at 65°C for 15 min. After recovering the supernatant containing DNA–RNA hybrids to a new tube, the beads were suspended in 40 µl of elution buffer again and incubated for an additional 10 min. The supernatants were combined into a single tube. After adding 97 µl of TE 10:1 and 3 µl of 20 mg/ml Proteinase K (Nacalai Tesque, 29442-85) to 100 µl of the supernatant or the input sample, the mixture was incubated at 50°C for 2 h. Two rounds of phenol/chloroform extraction and one round of chloroform extraction (Nacalai Tesque, 08402-55) were followed by ethanol precipitation. The precipitate was resuspended in TE 10:1.

### DRIP-sequencing analysis

We prepared a DNA library using KAPA HyperPrep Kit (KK8504) in combination with IDT for Illumina-TruSeq DNA UD Indexes Set A (Illumina, Inc., 20027213) for sequencing on the Illumina NovaSeq6000 platform with 101-bp paired-end reads. From 12 to 30 million mapped reads were obtained for each sequenced library (PRJDB20605). Raw reads were first trimmed to remove adapter sequences using cutadapt v2.7 [43]. After trimming, the reads <30 bp were discarded. The mean length of the remaining reads was 101 bp. The trimmed reads were then aligned to the *S. pombe* reference genome (ASM294v3) using Bowtie2 v2.3.5.1 with default parameters [44]. When a read matched a repetitive sequence, the read was evenly mapped to the repetitive region. DRIP-sequencing (DRIP-seq) peaks were called using MACS2 v2.2.6, with the --qvalue option set to 0.01, using input DNA sequencing reads as a control [45].

### Reverse transcription-quantitative PCR

5 × 10^8^ yeast cells were harvested from log-phase YE cultures, suspended in 400 µl of AE buffer (50 mM sodium acetate, pH 5.3, 10 mM EDTA), and stored at −80°C. After adding 40 µl of 10% SDS and 440 µl of phenol, the cell suspension was incubated at 65°C for 4 min. The solution was quickly chilled in a dry-ice/ethanol bath and then thawed by incubation at 65°C. RNA was purified using phenol/chloroform extraction. Four hundred microliters of the aqueous phase recovered was mixed with 40 µl of 3 M sodium acetate (pH 5.3) and 1 ml of 100% ethanol. After centrifugation at 17 900 × *g* at 4°C for 10 min, the pellet was rinsed with 1 ml of 80% ethanol and resuspended in 100 µl of TE 10:1. RNA concentrations were determined using NanoDrop One. Fifty microliters of RNA suspension was mixed with 125 µl of Monarch StabiLyse DNA/RNA buffer and further purified using Monarch spin RNA isolation kit (New England Biolabs, T2110). RNA concentrations were determined and adjusted to 1.0 µg/µl with RNase-free water. cDNA was synthesized from 1.0 µg of RNA using the LunaScript RT SuperMix kit (New England Biolabs, E3010), which contains random hexamer and poly (dT) primers. No-RT controls were used to detect DNA contamination. Details of quantitative PCR (qPCR) are explained below.

### Gross chromosomal rearrangement rates

A fluctuation assay determined spontaneous GCR rates [12, 20]. Yeast cells containing ChL were grown for 6–7 days on EMM plates supplemented with adenine and uracil (EMM+AU). With a single colony formed on the EMM+AU plates, 10 ml of EMM+AU liquid medium was inoculated. After 1–2 days of incubation, the cell culture was diluted in sterile deionized water and plated onto YNB and 5FOA plates supplemented with adenine and uracil (YNB+AU and 5FOA+AU, respectively). After 5–7 days, colonies formed on YNB+AU and 5FOA+AU plates were counted to determine the number of Leu+ and that of Leu+ Ura− cells, respectively. Leu+ Ura− colonies formed on 5FOA+AU were transferred to EMM plates supplemented with uracil (EMM+U) to examine adenine auxotrophy. The number of Leu+ Ura− Ade− cells indicative of GCR was determined by subtracting Leu+ Ura− Ade+ from Leu+ Ura− cells. The GCR rate per cell generation was determined as described previously [46]. At least 15 biologically independent experiments were performed for each strain (see the “Raw data”).

### Pulsed-field gel electrophoresis analysis of GCR products

From the parental (Leu+ Ura+ Ade+) and the GCR clones (Leu+ Ura− Ade−) obtained from biologically independent experiments, chromosomal DNAs were prepared in 1.6% low-melting agarose plugs (Nacalai Tesque, 01161-12) as described previously [39]. Chromosomal DNAs were separated in 0.55% certified megabase agarose (Bio-Rad, 1 613 109) using the CHEF-DRII system (Bio-Rad). For broad-rang pulsed-field gel electrophoresis (PFGE), chromosomal DNAs were resolved at 4°C in 1× TAE buffer (40 mM Tris-acetate, 1 mM EDTA) at 2 V/cm with a 1600 s pulse time for 42 h, followed by 2.4 V/cm with a 180 s pulse time for 4 h. For short-range PFGE, chromosomal DNAs were resolved at 4°C in 0.5× TBE buffer (89 mM Tris-borate, 2 mM EDTA) at 4.2 V/cm with a 60–100 s pulse time for 24 h. After electrophoresis, DNAs were stained with 0.2 µg/ml ethidium bromide (EtBr) (Nacalai Tesque, 14631-94) for 1 h and detected by Typhoon FLA9000 gel imaging scanner (GE Healthcare).

### Breakpoint analysis of GCR products

After PFGE, GCR products were recovered from the agarose gel using a FastGene Gel/PCR Extraction kit (Nippon Genetics, FG-91302). KOD FX Neo polymerase (Toyobo, KFX-201) and Q5 polymerase (New England Biolabs, M0491L) were used to amplify cnt3–imr3 junctions and irc3, respectively. PCR products were separated by 1.7% Seakem GTG agarose gel (Lonza, 50 070) electrophoresis in 1× TBE buffer, stained with 0.2 µg/ml of EtBr, and visualized using a Typhoon FLA9000 scanner.

### Fluorescent microscopy assay to detect Rad52-fmNeonGreen foci

Cells in log-phase EMM cultures were collected, stained with 2 µg/ml Hoechst 33 342 (Nacalai Tesque, 19172-51) at room temperature for 1 h in the dark, and placed on glass-bottom dishes (Matsunami Glass, D11130H). Fluorescence images were observed using the DeltaVision Personal fluorescence microscopy system (GE Healthcare), which is based on an Olympus wide-field IX71 fluorescence microscope equipped with a CoolSNAP HQ2 CCD camera (Photometrics) and an oil-immersion objective lens (UAPO 40×; NA = 1.35; Olympus). An exposure time of 0.8 s was used for fmNeonGreen. In each biologically independent experiment, >290 nuclei were counted. Images were processed using Fiji v2.16.0. Three biologically independent experiments were performed for each strain.

### Rad52 and Rad51 chromatin immunoprecipitation assay

ChIP experiments were performed, as previously described [47]. 1.5 × 10^8^ cells from log-phase EMM cultures were collected. After adding formaldehyde (Nacalai Tesque, 16223-55) to a final concentration of 1%, the cell suspension was vigorously mixed for 15 min at room temperature. After adding 3 ml of 2.5 M glycine to neutralize the crosslinker, the cell suspension was mixed for an additional 5 min. Cells were washed with 0.1% Lysis buffer (100 mM HEPES-KOH, pH 7.4, 140 mM NaCl, 1 mM EDTA, 0.1% Triton X-100, 0.1% Na-deoxycholate) and stored at −80°C. Cell pellets were resuspended in 200 µl of 0.1% Lysis buffer supplemented with 2 µl of protease inhibitor cocktail (Sigma, P8215) and 4 µl of 100 mM phenylmethanesulfonyl fluoride (PMSF) (Sigma, P7626). After adding an equal volume of acid-washed glass beads, cells were disrupted at 5000 rpm using a Micro Smash MS-100 (TOMY) for 30 s, 4 times with 3 min intervals on ice. We incubated 2 µl of anti-Flag M2 antibodies (Sigma–Aldrich, F1804) with 30 µl of Dynabeads M-280 sheep anti-Mouse IgG (Invitrogen, 11201D) in 400 µl of 1× PBS buffer supplemented with 2% bovine serum albumin at 4°C overnight. The beads were washed with 400 µl of 1% Lysis buffer and suspended with 340 µl of 1% Lysis buffer. After adding 60 µl of the cell extract, the bead suspension was incubated at 4°C for 2 h with rotation. Beads were washed and eluted as described in DRIP assays. Three biologically independent experiments were performed for each strain.

### qPCR analysis in DRIP-qPCR, RT-qPCR, and ChIP-qPCR

qPCR experiments in this work follow the MIQE guidelines [48]. qPCR was performed in 96-well plates (Bio-Bik, 3426-00) with sealing film (PlateSeal, qPCR pressure-activated sealing film, PS-PPO-100), in a StepOnePlus real-time PCR system (Applied Biosystems) with StepOne Software v2.3. Ten microliters of reaction volume per well. Holding stage at 95°C for 20 s. Cycling stage at 95°C for 3 s and then 60°C for 30 s, for 40 cycles. For reverse transcription-quantitative PCR (RT-qPCR) and ChIP-qPCR, we used Fast SYBR Green Master Mix (Thermo Fisher Scientific, 4 385 612). For DRIP-qPCR, we used PowerUp SYBR Green Master Mix (Thermo Fisher Scientific, A25742). The melting curve for each primer set was analyzed. Relative quantification of the samples was performed using a standard curve generated from serial dilutions of fission yeast genomic DNA. For a standard curve and RT-qPCR, three qPCR reactions (3× technical replicates) were set up for each target locus. For DRIP- and ChIP-qPCR, two qPCR reactions (2× technical replicates) were set up for each target locus. The mean of technical replicates was obtained. Details of qPCR data analyses, including mean, standard deviation (SD), *R*^2^, slope, y-intercept, PCR efficiency are shown in the “Raw data.” Three biologically independent experiments were performed for each strain (*n* = 3). qPCR primers were designed using Primer Express software (Applied Biosystems) or NCBI primer-BLAST [49] (Supplementary Table S2). Tm ≥ 54°C. Amplicon sizes were 80–260 bp.

### Western blot

Yeast cell extracts were prepared using the alkaline lysis method [50]. 1 × 10^8^ cells from log-phase YE cultures were collected, washed with H_2_O, and suspended in 300 µl H_2_O. After adding 300 µl of 0.6 M NaOH, the cell suspension was incubated at 30°C for 2.5 min with rotating. After centrifugation at 3200 × *g* for 2 min, cells were suspended in 140 µl of SDS sample buffer [60 mM Tris–HCl, pH 6.8, 4% β-mercaptoethanol, 4% SDS, 0.005% bromophenol blue (BPB), 5% glycerol] and incubated at 95°C for 3 min. After centrifugation at 17 900 × *g* for 1 min, cell extracts were recovered from the supernatant, separated by 10% SDS–polyacrylamide gel electrophoresis (SDS–PAGE) (acrylamide:bis-acrylamide = 29:1), and transferred onto a Polyscreen PVDF transfer membrane (Perkin Elmer, NEF1002001PK). The membrane was blocked in Blocking one (Nacalai Tesque, 03953-95) for 1 h and incubated with anti-Flag M2 primary antibodies (1:1000) at 4°C overnight. The membrane was incubated with peroxidase AffiniPure goat anti-mouse IgG (heavy + light) (Jackson ImmunoResearch Laboratories, 115-035-146) (1:10 000) secondary antibodies at 32°C for 1 h. The blot was developed using Supersignal West Femto substrate (Thermo Fisher Scientific, 34 094). Images were acquired using ImageQuant LAS500 (GE Healthcare).

### Rad52-6His3Flag protein expression and purification

Rad52-6His3Flag and Rad52-R45K-6His3Flag proteins were expressed in *E. coli* strain BL21-CodonPlus (DE3)-RIPL using plasmids pTN1287 and pTN1298, respectively. Cells were grown in 500 ml of LB medium (Lennox) (10 g/l tryptone, 5 g/l YE, 5 g/l NaCl) supplemented with 50 µg/ml of ampicillin at 30°C. When the optical density at 600 nm reached ~0.5, 1 M of isopropyl-β-d-thiogalactopyranoside (IPTG) (Nacalai Tesque, 19742-94) was added to a final concentration of 1 mM. After 3-h incubation, the cells were collected by centrifugation at 6000 × *g* for 10 min at 4°C and stored at −80°C. Cells were resuspended in 20 ml of buffer R (20 mM Tris–HCl, pH 8.0, 500 mM NaCl, 1 mM EDTA, 10% glycerol, and 1 mM dithiothreitol (DTT)) supplemented with 1 mM PMSF and 2 mM benzamidine, and disrupted by nine rounds of 20 s sonication using a Sonifier 250. After centrifugation at 40 000 × *g* for 30 min at 4°C, the supernatant was mixed with an equal volume of buffer R containing 60% ammonium sulfate (Nacalai Tesque, 02620-75). The mixture was stirred at 4°C for 30 min. After centrifugation at 20 000 × *g* for 20 min at 4°C, the precipitate was recovered and suspended in 5 ml of binding buffer (20 mM NaH_2_PO_4_, pH 8.0, 400 mM NaCl, 10% glycerol, 10 mM imidazole, 0.1% Triton X-100). The protein solution was applied to a column containing 1.5 ml of TALON metal affinity resin (Takara, 635 502) to capture the His-tagged Rad52 protein. The column was washed three times with 5 ml of Washing buffer (20 mM NaH_2_PO_4_, pH 8.0, 400 mM NaCl, 20 mM imidazole, 0.1% TritonX-100), and the Rad52 protein was eluted in 0.5 ml fractions of elution buffer (20 mM NaH_2_PO_4_, pH 8.0, 400 mM NaCl, 10% glycerol, 200 mM imidazole, 0.1% TritonX-100). The second and third elution fractions were combined and dialyzed against Storage buffer (20 mM Tris–HCl, pH 7.5, 1 mM EDTA, 175 mM NaCl, 10% glycerol, and 1 mM DTT) using Dialysis membrane size 8 (FujiFilm, 046-30 911) and stored at −80°C.

### Rad51 protein expression and purification

Rad51 protein was expressed and purified as described previously [51]. In brief, the Rad51 protein was expressed in *E. coli* BL21-CodonPlus (DE3)-RIPL using pET11b, with 1 mM IPTG at 18°C for 12 h. Cells were disrupted by sonication, and the whole cell extract was clarified by centrifugation at 70 000 × *g* for 1 h. The supernatant was mixed with ammonium sulfate to 35% saturation and centrifuged at 10 000 × *g* for 30 min. The precipitate was resuspended in P buffer (20 mM KH_2_PO_4_, pH 7.4, 0.5 mM EDTA, 10% glycerol, 1 mM DTT) and applied to SP Sepharose (GE Healthcare). The flow-through fraction was then applied to Q Sepharose (GE Healthcare) and eluted with a gradient from 100 to 800 mM KCl. Combined peak fractions were applied to a HiTrap Heparin column, and Rad51 was eluted with a gradient from 100 to 700 mM KCl. The combined peak fractions were then applied to Resource Q (GE Healthcare) and eluted with a gradient from 100 to 600 mM KCl. Peak fractions were combined, dialyzed against P buffer containing 200 mM KCl, and concentrated using Amicon Ultra-4 (MWCO 10 000). The aliquoted samples were frozen in liquid nitrogen and stored at −80°C.

### Preparation of nucleic acid substrates

To prepare R-loop, D-loop, DNA bubble, and dsDNA substrates, oligonucleotides listed in Supplementary Table S2 were mixed at a final concentration of 625 nM in Duplex buffer (100 mM potassium acetate, 30 mM HEPES, pH 7.5), denatured at 95°C for 2 min, and annealed by gradually reducing the temperature (−0.5°C per 30 s) to 4°C using the thermal cycler T100 (Bio-Rad) [52]. The formation of the substrates was confirmed by 3% agarose gel (Nacalai Tesque, 01153-22) electrophoresis in 1× TBE buffer.

### Rad52- and Rad51-mediated annealing assay

To label 5′ termini of C1 or nC1 oligonucleotides (oligos) with ^32^P, 5 pmol of C1 or nC1 oligos, 5 pmol of γ-^32^P-ATP (Revvity, BLU002A, 3000 Ci/mmol), and 1 µl of 10 unit/µl T4 polynucleotide kinase (New England Biolabs, M0201S) in 1× T4 PNK buffer were incubated at 37°C for 30 min. The ^32^P-labeled C1 or nC1 oligos (0.3 nM in DNA molecules) and Rad52 proteins (1.35 nM) or Rad51 proteins (4.5 nM) were incubated in 200 µl of Annealing buffer (25 mM Tris-acetate, pH 7.5, 1 mM MgCl_2_, 100 µg/ml bovine serum albumin, 1 mM DTT, in diethylpyrocarbonate-treated H_2_O) at 30°C for 10 min. 0.6 µl of 100 nM substrates in Annealing buffer was added to the reaction, resulting in a final concentration of 0.3 nM. Twenty microliter aliquots were withdrawn at the indicated time and mixed with 20 µl of 2× Stop buffer (3% SDS, 0.1% BPB, 30 nM cold C1 or nC1 oligos). After adding 4 µl of Proteinase K solution (Nacalai Tesque, 15679-64), the samples were incubated at 37°C for 1 h and loaded onto 8% non-denaturing PAGE in 1× TBE buffer at 10 V/cm for 50 min. Gels were dried on DE81 ion exchange cellulose chromatography paper (Whatman, 3658-915) using a vacuum gel-drying apparatus at 65°C for 45 min. Radioactive signals were detected using a phosphorimager Typhoon FLA7000 (GE Healthcare) and quantified with Multi Gauge v3.2.

### Statistics

A two-tailed Mann–Whitney test and a two-tailed Student *t*-test were performed with GraphPad Prism v10.4.1 for macOS and Microsoft Excel (version 16), respectively. For DRIP-qPCR, a two-tailed Student *t*-test was performed between RNaseH “−” samples of wild type and mutant strains, as well as the indicated pairs of mutant strains. For ChIP-qPCR, RT-qPCR, and Rad52 focus formation, a two-tailed Student *t*-test was performed between wild type and mutant strains, as well as the indicated pairs of mutant strains. For GCR rates, a two-tailed Mann–Whitney test was performed between wild type and mutant strains as well as the indicated pairs of mutant strains. *P < .05, **P < .01, ^****^P < .001, ^*****^P < .0001, ns = not significant.

## Results

### Heterochromatin suppresses R-loop formation at pericentromeric repeats via transcriptional silencing

**Figure 1.**
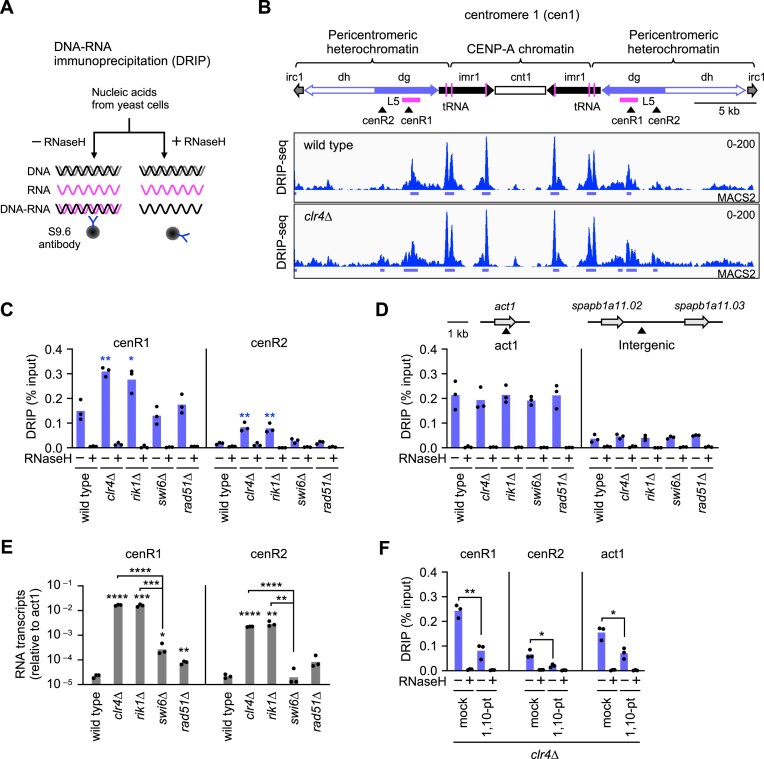
Clr4 methyltransferase suppresses DNA–RNA hybrid formation at pericentromeric repeats. (**A**) Schematic of DRIP assay. Nucleic acids extracted from yeast cells were sonicated and incubated in the presence or absence of RNaseH. DNA–RNA hybrids were immunoprecipitated using the S9.6 antibody. (**B**) DRIP-seq data of wild type and *clr4∆* strains in centromere 1 (cen1). Centromere inverted repeats (imr, dg, dh, and irc) flank the central unique sequence (cnt). In contrast to humans, there are no tandem repeats at the CENP-A domain in *S. pombe*. Vertical magenta bars indicate positions of tRNA genes. Arrowheads indicate DRIP-qPCR amplification sites. MACS2 indicates the regions where DNA–RNA hybrids are significantly accumulated. (**C, D**) DRIP-qPCR. DNA–RNA hybrid levels at (C) centromeric cenR1 and cenR2 sites and (D) non-centromeric act1 and Intergenic sites in wild type, *clr4∆, rik1∆, swi6∆*, and *rad51∆* strains. DRIP-qPCR amplification sites of *act1* and Intergenic are indicated at the top of the graph. The percent recovery is shown. Each dot represents a biologically independent experiment (*n* = 3). Bars show the mean. (**E**) RT-qPCR. RNA transcript levels of cenR1 and cenR2 were normalized relative to act1 RNA levels. (**F**) Treatment of *clr4∆* cells with a transcription inhibitor, 1,10-phenanthroline (1,10-pt) reduced DNA–RNA hybrids.

To determine whether loss of H3K9me2/3 increases R-loop formation at pericentromeric repeats, we performed DRIP assays using the wild type and *clr4∆* strains of fission yeast. Nucleic acids were prepared from yeast cells, sonicated into fragments, and DNA–RNA hybrids were immunoprecipitated using the S9.6 antibody, which captures DNA–RNA hybrids (Fig. 1A) [53]. Fission yeast centromeres consist of the central unique sequence (cnt) surrounded by inverted repeats (imr, dg, dh, and irc) (Fig. 1B, top). The CENP-A chromatin forms on the cnt and an inner part of imr repeats. Pericentromeric heterochromatin marked by H3K9me2/3 flanks the central CENP-A chromatin domain. DRIP followed by deep sequencing (DRIP-seq) detected DNA–RNA hybrids at tranfer RNA (tRNA) genes (Fig. 1B, vertical magenta lines), consistent with previous reports [29]. The L5 fragment is known to induce heterochromatin formation at ectopic sites outside centromeres [54]. In agreement with the role of DNA–RNA hybrids in promoting heterochromatin assembly [55], the L5 region showed hybrid accumulation. Overall levels of DNA–RNA hybrids at centromeres appeared similar in wild type and *clr4∆* cells (Fig. 1B). However, peak calling using MACS2 [45] (see the “Materials and methods” section) identified two regions in the pericentromere, designated cenR1 and cenR2, where DNA–RNA hybrids were enriched in *clr4∆* cells (Fig. 1B). *clr4∆* increased DNA–RNA hybrid levels not only in cen1 but also in cen2 and cen3 (Supplementary Fig. S1A, see below). DRIP followed by qPCR (DRIP-qPCR) confirmed increased DNA–RNA hybrids at cenR1 and cenR2 in *clr4∆* cells (Fig. 1C). The S9.6 antibody can bind double-stranded RNA in addition to DNA–RNA hybrids [56, 57]. However, double-stranded RNA should not be detected in our assays because we performed qPCR or deep sequencing without reverse transcription. Treatment of nucleic acids with *E. coli* RNaseH prior to immunoprecipitation eliminated the DRIP-qPCR signals, confirming that our DRIP assay specifically detected DNA–RNA hybrids. In contrast to cenR1 and cenR2, *clr4∆* did not significantly change the hybrid levels at tRNA and cnt1 in the centromere (Supplementary Fig. S1B–D) and *act1* and an intergenic site outside the centromere (Fig. 1D). These results suggest that loss of H3K9me2/3 increases R-loops at specific sites in pericentromeric repeats.

**Figure 2.**
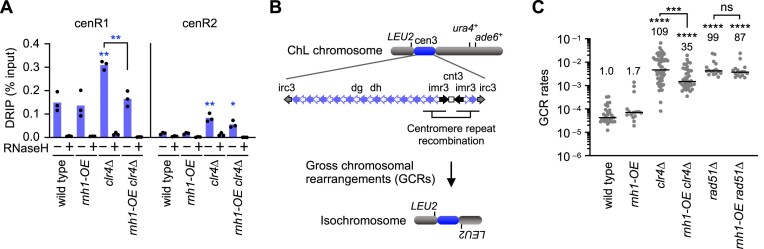
DNA–RNA hybrid accumulation at pericentromeric repeats causes GCRs. (**A**) Rnh1 overexpression reduced DNA–RNA hybrids in *clr4∆* cells. DNA–RNA hybrid levels at cenR1 and cenR2 in wild type, *rnh1-OE, clr4∆*, and *rnh1-OE clr4∆* strains. Each dot represents a biologically independent experiment (*n* = 3). Bars show the mean. (**B**) Illustrated are the extra-chromosome, ChL, and the centromere repeats in cen3. GCRs losing *ura4*+ and *ade6*+ markers were detected. Isochromosomes whose arms are mirror images are the major GCR products formed in *clr4∆* or *rad51∆* cells. (**C**) Rnh1 overexpression reduced GCRs in *clr4∆* cells. GCR rates of wild type, *rnh1-OE, clr4∆, rnh1-OE clr4∆, rad51∆*, and *rnh1-OE rad51∆* strains. Each dot represents a biologically independent experiment. Lines show the median. GCR rates relative to wild type are shown at the top of each column.

To further explore this effect, we analyzed *rik1∆* and *swi6∆* cells. Rik1 interacts with Clr4 and facilitates H3K9me2/3 at pericentromeric repeats [58–60]. Swi6, the fission yeast homolog of heterochromatin protein 1 (HP1), binds to H3K9me2/3 and enforces sister centromere cohesion [61, 62]. In contrast to Clr4, Swi6 is not essential for H3K9me2/3 or transcriptional silencing at pericentromeric repeats [63, 64]. Like *clr4∆, rik1∆* increased DNA–RNA hybrids at cenR1 and cenR2, whereas *swi6∆* did not (Fig. 1C and D). Loss of the key player of homologous recombination (HR) Rad51 increases isochromosome formation [12, 65] (Fig. 2C). However, unlike *clr4∆, rad51∆* did not significantly increase DNA–RNA hybrids, suggesting that R-loops are not a byproduct of GCRs. These data suggest that H3K9me2/3-mediated transcriptional silencing is crucial for suppressing R-loop accumulation.

To determine transcriptional levels at cenR1 and cenR2, we prepared total RNA from yeast cells and performed reverse transcription followed by qPCR, RT-qPCR (Fig. 1E). As expected, essentially no qPCR amplification was observed when reverse transcription was omitted (Supplementary Fig. S1E). *clr4∆* and *rik1∆* strongly increased cenR1 and cenR2 transcripts, while *swi6∆* and *rad51∆* only slightly increased cenR1 transcripts, suggesting a link between transcription and R-loop accumulation. As a complementary experiment, we forced-reduced transcription using a transcription inhibitor and determined its effect on DNA–RNA hybrid levels by DRIP-qPCR (Fig. 1F). Compared with a mock treatment, treatment of *clr4∆* cells with the transcription inhibitor 1,10-phenanthroline (1,10-pt) reduced DNA–RNA hybrids [66, 67]. Together, these results demonstrate that heterochromatin suppresses R-loop accumulation at pericentromeric repeats via transcriptional silencing.

### Loss of heterochromatin causes GCRs through R-loop accumulation

As *clr4∆* and *rik1∆*, but not *swi6∆*, increase isochromosome formation [13, 20], R-loops might cause centromeric GCRs. To test this possibility, we overexpressed the yeast RNaseH1 homolog Rnh1 [68] under a strong *adh1* promoter from an ectopic chromosomal locus. DRIP-qPCR showed that the Rnh1 overexpression (*rnh1-OE*) did not significantly change DNA–RNA hybrid levels in wild-type cells. However, in *clr4∆* cells, *rnh1-OE* reduced the hybrid levels at cenR1 (Fig. 2A and Supplementary Fig. S2), showing that DNA–RNA hybrids accumulated in *clr4∆* cells are hypersensitive to Rnh1. We next assessed whether *rnh1-OE* also affects GCR rates. For this purpose, we employed a previously established GCR assay using the extra-chromosome ChL, derived from chr3 (Fig. 2B) [12]. Like cen1, cen3 contains cenR1 and cenR2 sequences in pericentromeric repeats (Fig. 1 and Supplementary Fig. S1A). In this assay, yeast cells harboring ChL were grown in media supplemented with uracil and adenine, and those that had undergone GCRs that resulted in loss of *ura4*+ and *ade6*+ marker genes were detected using selection plates (see the “Materials and methods” section). Because ChL is dispensable for cell viability, we can detect otherwise lethal GCRs, such as isochromosome formation, in haploid cells. Fluctuation tests showed that *clr4∆* significantly increased GCR rates (Fig. 2C), consistent with previous reports [20]. Importantly, *rnh1-OE* reduced GCR rates specifically in *clr4∆* cells, mirroring its effect on DNA–RNA hybrid levels. In contrast to *clr4∆* cells, *rnh1-OE* had no significant effect on GCR rates in *rad51∆* cells, which do not accumulate DNA–RNA hybrids at pericentromeric repeats (Fig. 1C), supporting the DNA–RNA hybrid-mediated effect on GCRs. Further reinforcing the role of R-loops in centromeric GCRs, loss of both *rnh1*+ and *rnh201*+ genes, encoding RNaseH1 and RNaseH2, respectively, increased isochromosome formation (Supplementary Fig. S3). Together, these results demonstrate that loss of heterochromatin causes GCRs by accumulating R-loops at pericentromeric repeats.

### Transcriptional PBR cycles accumulate R-loops at pericentromeric repeats

**Figure 3.**
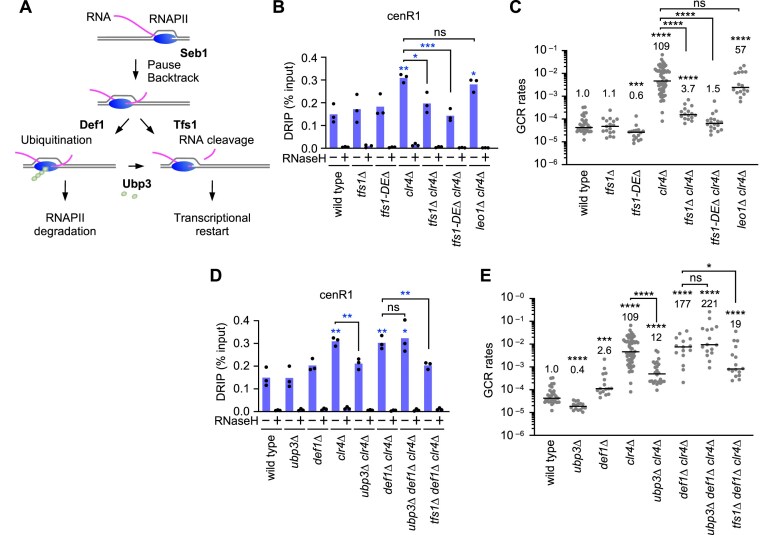
Tfs1 and Ubp3 promote DNA–RNA hybrid accumulation and cause GCRs in *clr4∆* cells. (**A**) Illustrated are the roles of Tfs1, Def1, Ubp3, and Seb1. Tfs1 facilitates transcriptional restart by promoting RNA cleavage by RNAPII. Def1 promotes ubiquitin-dependent RNAPII degradation, whereas Ubp3 prevents RNAPII degradation by removing ubiquitin from RNAPII. Seb1 causes transcriptional pausing at pericentromeric repeats. (**B**) DNA–RNA hybrid levels at cenR1 and (**C**) GCR rates in wild type, *tfs1∆, tfs1-DE∆, clr4∆, tfs1∆ clr4∆, tfs1-DE∆ clr4∆*, and *leo1∆ clr4∆* strains. (**D**) DNA–RNA hybrid levels at cenR1 and (**E**) GCR rates in wild type, *ubp3∆, def1∆, clr4∆, ubp3∆ clr4∆, def1∆ clr4∆, ubp3∆ def1∆ clr4∆*, and *tfs1∆ def1∆ clr4∆* strains. (B, D) Each dot represents a biologically independent experiment (*n* = 3). Bars show the mean. (C, E) Each dot represents a biologically independent experiment. Lines show the median. GCR rates relative to wild type are shown at the top of each column.

Previously, we reported that Tfs1/TFIIS and Ubp3, which facilitate transcriptional restart (Fig. 3A), are required for GCRs to occur in *clr4∆* cells [19, 20]. These findings suggest that transcriptional restart contributes to R-loop accumulation. Indeed, DRIP-qPCR showed that *tfs1∆* reduced R-loops at cenR1 and cenR2 in *clr4∆* cells (Fig. 3B and Supplementary Fig. S4A). Elimination of the acidic residues, D274 and E275, of Tfs1, specifically required to stimulate Rpb1’s RNA cleavage activity [69, 70], *tfs1-DE∆*, also reduced both R-loops and GCRs in *clr4∆* cells (Fig. 3B and C, and Supplementary Fig. S4A). Tfs1 also interacts with the PAF1 complex, which supports transcription elongation [71]. However, loss of Leo1, a component of the PAF1 complex, did not significantly change R-loop or GCR levels in *clr4∆* cells (Fig. 3B and C). These results suggest that Tfs1 facilitates the accumulation of genotoxic R-loops by promoting transcriptional restart.

Transcriptional pausing can result in ubiquitin-dependent degradation of RNAPII (Fig. 3A) [27]. Degradation factor 1, Def1, promotes ubiquitin-dependent degradation of Rpb1 [72], whereas a ubiquitin protease, Ubp3, counteracts this by deubiquitinating Rpb1, thereby promoting transcriptional restart [28]. We examined whether Ubp3 also facilitates R-loop accumulation. In *clr4∆* cells, *ubp3∆* reduced R-loops at cenR1 (Fig. 3D and Supplementary Fig. S4B). However, in *def1∆ clr4∆* cells, *ubp3∆* did not significantly change R-loop or GCR levels (Fig. 3D and E), showing that Ubp3 antagonizes Def1 to promote R-loop accumulation and GCRs. Notably, even in *def1∆ clr4∆* cells, *tfs1∆* reduced both R-loops and GCRs (Fig. 3D and E), underscoring the essential role of Tfs1 in genotoxic R-loop accumulation. Importantly, *rnh1-OE* reduced GCR rates in *clr4∆* cells but not in *tfs1∆ clr4∆* or *ubp3∆ clr4∆* cells (Supplementary Fig. S4C). These results show that, when heterochromatin is lost, Tfs1 and Ubp3 promote the accumulation of genotoxic R-loops at pericentromeric repeats, probably by promoting transcriptional restart.

**Figure 4.**
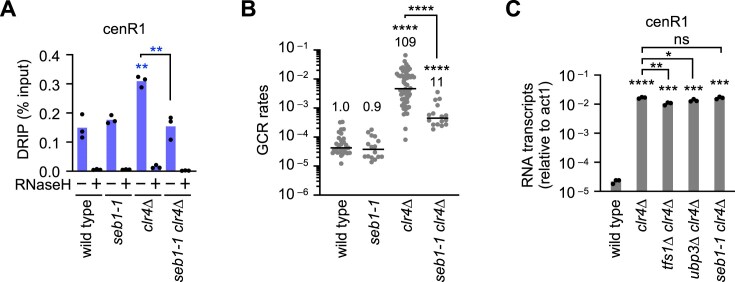
Seb1 promotes DNA–RNA hybrid accumulation and causes GCRs in *clr4∆* cells. (**A**) DNA–RNA hybrid levels at cenR1 in wild type, *seb1-1, clr4∆*, and *seb1-1 clr4∆* strains. Each dot represents a biologically independent experiment (*n* = 3). Bars show the mean. (**B**) GCR rates in wild type, *seb1-1, clr4∆*, and *seb1-1 clr4∆* strains. Each dot represents a biologically independent experiment. Lines show the median. GCR rates relative to wild type are shown at the top of each column. (**C**) RT-qPCR. RNA transcript levels of cenR1 relative to act1 are shown in wild type, *clr4∆, tfs1∆ clr4∆, ubp3∆ clr4∆*, and *seb1-1 clr4∆* strains. Each dot represents a biologically independent experiment (*n* = 3). Bars show the mean.

Seb1, an essential transcription termination factor, induces transcriptional pausing at pericentromeric repeats and facilitates heterochromatin assembly (Fig. 3A) [22–24]. We hypothesized that transcriptional restart following Seb1-mediated pausing facilitates R-loop accumulation. To test this, we introduced the *seb1-1* mutation, which specifically impairs the pausing [24], into *clr4∆* and wild-type cells. *seb1-1* reduced R-loops at cenR1 and GCR rates in *clr4∆* but not in wild-type cells (Fig. 4A and B, and Supplementary Fig. S4D). RT-qPCR showed that neither *tfs1∆, ubp3∆*, nor *seb1-1* eliminated pericentromeric RNA in *clr4∆* cells (Fig. 4C and Supplementary Fig. S4E and F). *tfs1∆* and *ubp3∆* only slightly reduced cenR1 and cenR2 transcripts in *clr4∆* cells, and *seb1-1* did not significantly change the RNA level. In *seb1-1 clr4∆* cells, non-PBR transcription might compensate for the lack of PBR cycles to maintain the transcript level. These findings demonstrate that the transcriptional PBR cycle, but not transcription in general, accumulates genotoxic R-loops at pericentromeric repeats when heterochromatin is lost.

### Rad52, but not Rad51, causes GCRs in the absence of heterochromatin

**Figure 5.**
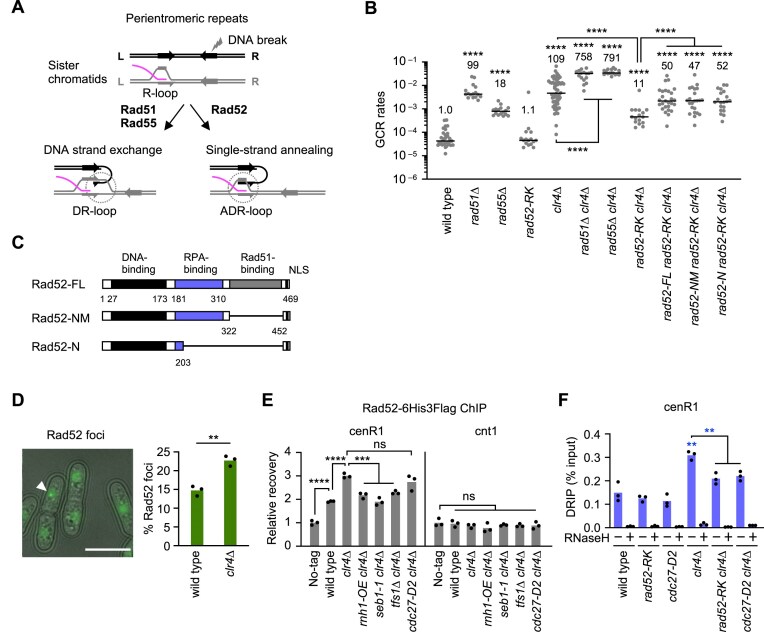
Rad52 causes GCRs in *clr4∆* cells. (**A**) Homology-mediated recombination with R-loops. Rad51 promotes DNA strand exchange near, but not at, R-loops to form DR-loops. Rad52 promotes SSA with ssDNA region within R-loops to form ADR-loops. (**B**) GCR rates of wild type, *rad51∆, rad55∆, rad52-R45K, clr4∆, rad51∆ clr4∆, rad55∆ clr4∆, rad52-R45K clr4∆, rad52-FL rad52-R45K clr4∆, rad52-NM rad52-R45K clr4∆*, and *rad52-N rad52-R45K clr4∆* strains. Each dot represents a biologically independent experiment. Lines show the median. GCR rates relative to wild type are shown at the top of each column. (**C**) Rad52-FL, Rad52-NM, and Rad52-N were expressed at an ectopic site on chr2. (**D**) Clr4 suppresses Rad52 focus formation. The image shows Rad52-fmNeonGreen foci in wild-type cells. Fluorescence and DIC images are overlaid. DIC, differential interference contrast. An arrow indicates Rad52 focus. A bar, 10 µm. A bar graph shows the percentage of cells containing Rad52-fmNeonGreen foci. Each dot represents an independent experiment. Bars show the mean. (**E**) Rad52 localization at pericentromeric repeats. Rad52-6His3Flag ChIP experiments using no-tag strain and *rad52-6His3Flag* strains of wild type, *clr4∆, rnh1-OE clr4∆, seb1-1 clr4∆, tfs1∆ clr4∆*, and *cdc27-D2 clr4∆*. The recovery relative to the no-tag control is shown. Each dot represents an independent experiment (*n* = 3). Bars show the mean. (**F**) Rad52 and Cdc27 stabilize DNA–RNA hybrids at pericentromeric repeats. DNA–RNA hybrid levels at cenR1 in wild type, *rad52-R45K, cdc27-D2, clr4∆, rad52-R45K clr4∆*, and *cdc27-D2 clr4∆* cells. Each dot represents an independent experiment (*n* = 3). Bars show the mean.

How do R-loops cause GCRs? It has been reported that *clr4∆* increases isochromosome formation through recombination between centromeric inverted repeats [20]. Yeast has Rad51-dependent and Rad51-independent pathways of homology-mediated recombination (Fig. 5A). In the Rad51-dependent pathway, Rad51 binds ssDNA and mediates DNA strand exchange with a homologous duplex [73]. Rad55, the fission yeast homolog of human Rad51C [74], supports Rad51-dependent recombination. R-loops can promote Rad51-dependent strand exchange, forming structures known as DR-loops (Fig. 5A, left) [75]. In this case, Rad51 facilitates DNA strand exchange near R-loops but not at R-loops. The Rad51-independent pathway relies on Rad52, which binds ssDNA and promotes SSA between homologous sequences. In contrast to Rad51, Rad52-dependent SSA might utilize the displaced ssDNA region within R-loops to form ADR-loops (Fig. 5A, right). To determine which pathway is responsible for GCRs in the absence of heterochromatin, we introduced *rad51, rad55*, or *rad52* mutations into yeast cells and determined GCR rates (Fig. 5B). *rad51∆* or *rad55∆* increased GCR rates in both wild type and *clr4∆* cells, showing that Rad51 and Rad55 suppress, rather than promote, GCRs. In contrast to *rad51∆* and *rad55∆*, the *rad52-R45K* mutation, which specifically impairs SSA activity [37, 76], reduced GCR rates in *clr4∆* cells, suggesting that Rad52-dependent SSA is responsible for GCRs when heterochromatin is lost. Importantly, *rad52-R45K* did not further reduce GCR rates in *tfs1∆ clr4∆* cells (Supplementary Fig. S5A), indicating that Rad52 and Tfs1 act in the same pathway. Rad52 contains several functional domains, including DNA-binding, Replication protein A (RPA)-binding, Rad51-binding domains, and a nuclear localization sequence (NLS) (Fig. 5C). The RPA-binding and Rad51-binding domains are dispensable for SSA [77]. Ectopic expression of Rad52 full-length (Rad52-FL) or the truncated forms (Rad52-NM or Rad52-N) restored GCR rates to similar levels in *rad52-R45K clr4∆* cells, demonstrating that the RPA- and Rad51-binding domains are dispensable for GCRs. These results indicate that R-loops cause GCRs via Rad52-dependent SSA but not through Rad51-dependent HR.

Rad52 proteins accumulate at sites of DNA repair or recombination, forming nuclear foci [78]. To assess whether heterochromatin affects Rad52 focus formation, we tagged Rad52 with fmNeonGreen at its endogenous locus and observed the Rad52 focus using fluorescence microscopy (Fig. 5D). The fraction of cells exhibiting Rad52 foci increased in *clr4∆* cells, indicating that heterochromatin suppresses Rad52 focus formation. To further investigate whether Rad52 is recruited to pericentromeric repeats, we expressed Rad52-6His3Flag [37] from its endogenous locus and performed ChIP using an anti-Flag M2 antibody. ChIP-qPCR revealed that *clr4∆* increased Rad52 localization at cenR1 but not at cnt1 or act1 (Fig. 5E and Supplementary Fig. S5B). Either *rnh1-OE, seb1-1*, or *tfs1∆* reduced the Rad52 localization at cenR1. In contrast to Rad52, *clr4∆* did not significantly increase Rad51 localization at cenR1 (Supplementary Fig. S6). These results suggest that loss of heterochromatin specifically increases Rad52 localization at pericentromeric repeats in a manner that depends on R-loops produced by PBR cycles. Interestingly, DRIP-qPCR showed that *rad52-R45K* reduced DNA–RNA hybrids at cenR1 in *clr4∆* cells (Fig. 5F and Supplementary Fig. S5C), suggesting that Rad52 stabilizes DNA–RNA hybrids by converting R-loops into ADR-loops.

### The Rad52 protein forms ADR-loops *in vitro*

**Figure 6.**
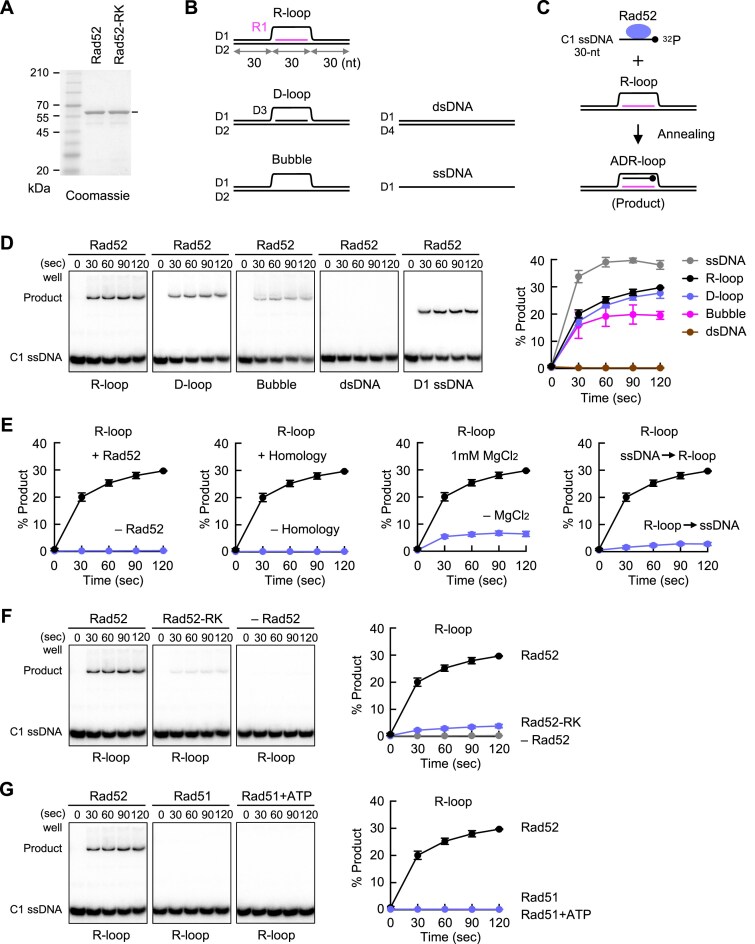
Rad52 protein converts R-loops into ADR-loops. (**A**) Purified wild-type Rad52 and mutant Rad52-R45K proteins were separated by 10% SDS–PAGE and stained with Coomassie brilliant blue. Sizes of CLEARLY Stained Protein Ladder (Takara) are indicated on the left of the panel. (**B**) Illustrated are the substrates used in this assay. (**C**) Schematic of the reaction. Rad52 was pre-incubated with C1 ssDNA labeled with ^32^P at the 5′-end (a black circle). The reaction was initiated by adding substrates, such as the R-loop. The reaction mixture contains 0.3 nM of C1, 0.3 nM of substrates, and 1.35 nM of Rad52. (**D**) The reaction product was separated by 8% non-denaturing PAGE in 1× TBE buffer. Radiation signals were detected using a phosphorimager FLA7000. Percentages of annealing products over time are shown in the graph. Mean ± SD of three independent experiments. (**E**) The ADR-loop formation under different conditions. In “–Homology,” instead of C1, nC1 ssDNA that is not complementary to D1 was used. In “R-loop→ssDNA,” Rad52 was pre-incubated with R-loops before adding C1 ssDNA. (**F**) Rad52-R45K hardly promotes ADR-loop formation. (**G**) The ADR-loop formation assay using Rad51 instead of Rad52. Reactions were performed using 4.5 nM Rad51 in the absence or presence of 1 mM ATP.

To test whether Rad52 forms ADR-loops (Fig. 5A), we performed an *in vitro* assay using Rad52-6His3Flag protein, expressed in *E. coli* and purified using TALON metal affinity resin, which binds His-tagged proteins (Fig. 6A and Supplementary Fig. S7A). We prepared five types of nucleic acid substrates (Fig. 6B, RNA shown in magenta; Supplementary Fig. S7B). All substrates contain 90-nt D1 oligo, whose central region is complementary to 30-nt C1 oligo (Fig. 6C). D1 and D2 oligos are mostly complementary, except for a central 30-nt region. R-loops and D-loops were assembled using 30-nt R1 RNA or D3 DNA, respectively. In the assay, Rad52 protein was pre-incubated with ^32^P-labeled C1 at 30°C for 10 min, followed by the addition of a substrate (Fig. 6C, R-loop is depicted). At indicated time points, samples were taken, deproteinized, and analyzed by native PAGE (Fig. 6D). Phosphorimager analysis showed that 20% of C1 annealed to the R-loop within the first 30 s, and ~30% formed ADR-loops within 120 s (Fig. 6D, black circles in the graph). Both R-loops and D-loops were more effective substrates than “Bubbles” but not as efficient as D1 “ssDNA.” During the reaction, the RNA (R1) and DNA (D3) components remained hybridized to D2 in the R-loops and D-loops, respectively (Supplementary Fig. S7C). No strand annealing occurred with “dsDNA,” indicating that Rad52-dependent annealing requires single-stranded regions. We confirmed that ADR-loop formation requires Rad52 and sequence complementarity between the probe and the R-loop (Fig. 6E). Adding 1 mM MgCl_2_ enhanced ADR-loop formation, likely by increasing Rad52’s DNA-binding activity [79]. Pre-mixing Rad52 with R-loops before adding the C1 probe resulted in minimal ADR-loop formation. Importantly, the Rad52-R45K mutant protein formed ADR-loops at ~10-fold lower levels than the wild-type Rad52 after the first 30 s (Fig. 6F). In contrast to Rad52, the Rad51 protein [51] did not promote ADR-loop formation either in the absence or presence of ATP under the experimental condition we used (Fig. 6G). Even when we used D-loop or D1 ssDNA substrates, Rad51 did not form annealing products (Supplementary Fig. S8) while it facilitates DNA stand exchange [51]. These biochemical data demonstrate that the Rad52 protein specifically converts R-loops into ADR-loops via SSA.

### Polδ-dependent BIR causes GCRs when ADR-loops are formed

**Figure 7.**
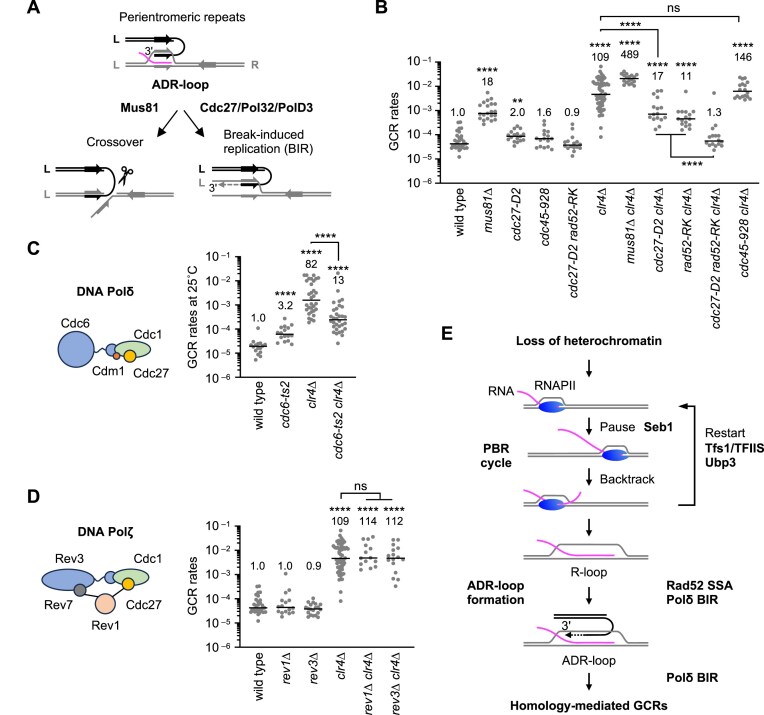
DNA Polδ causes GCRs in *clr4∆* cells. (**A**) Following ADR-loop formation, crossover or BIR between pericentromeric inverted repeats can form isochromosomes. L, left; R, right. (**B**) Cdc27 is required for GCRs in *clr4∆* cells. GCR rates of wild type, *mus81∆, cdc27-D2, cdc45-928, cdc27-D2 rad52-R45K, clr4∆, mus81∆ clr4∆, cdc27-D2 clr4∆, rad52-R45K clr4∆, cdc27-D2 rad52-R45K clr4∆*, and *cdc45-928 clr4∆* strains. (**C**) Cdc6, the catalytic subunit of Polδ, is required for GCRs in *clr4∆* cells. GCR rates of wild type, *cdc6-ts2, clr4∆*, and *cdc6-ts2 clr4∆* strains at 25°C. (**D**) Rev1 and Rev7, subunits of Polζ, are dispensable for GCRs. GCR rates of wild type, *rev1∆, rev3∆, clr4∆, rev1∆ clr4∆*, and *rev3∆ clr4∆* strains. Each dot represents a biologically independent experiment. Lines show the median. GCR rates relative to wild type are shown at the top of each column. (**E**) A model in which transcriptional PBR cycles form R-loops and Rad52 converts R-loops into ADR-loops, resulting in homology-mediated GCRs. Seb1 causes transcription pausing at pericentromeric repeats. Tfs1/TFIIS and Ubp3 promote transcriptional restart. Rad52 forms ADR-loops from R-loops and ssDNA, bridging a pair of pericentromeric inverted repeats. Polδ initiates BIR at the ADR loop, resulting in the isochromosome formation.

Once ADR-loops are formed between pericentromeric inverted repeats, they can lead to isochromosome formation through crossover or BIR (Fig. 7A). The Mus81 endonuclease promotes crossover rather than non-crossover recombination [80], whereas Polδ is essential for BIR and chromosomal replication [81]. Previous studies have shown that, in *rad51∆* cells, Mus81, but not the Polδ subunit Cdc27 (also known as Pol32/PolD3), is required for the spontaneous formation of isochromosomes [65], showing that crossover recombination produces isochromosomes in the absence of Rad51. However, in *clr4∆* cells, mutation of *cdc27* [82] reduced GCR rates, while *mus81∆* did not (Fig. 7B and Supplementary Fig. S9), suggesting that BIR rather than crossover is the mechanism of isochromosome formation when heterochromatin is lost and ADR-loops are produced. Notably, *cdc27-D2* cells do not exhibit temperature-sensitive growth defects [82], indicating that chromosomal replication remains largely intact. The CMG helicase, which contains Cdc45, Mcm2-7, and GINS, is essential for chromosomal replication [83]. To further assess whether chromosomal replication contributes to GCRs, we examined the *cdc45-928* mutation, which partially impairs DNA replication at 30°C [84], and found that it did not reduce GCR rates in *clr4∆* cells (Fig. 7B). These results support the notion that Cdc27 promotes GCRs via BIR, rather than through chromosomal replication. Like *rad52-R45K, cdc27-D2* did not further reduce GCRs in *tfs1∆ clr4∆* cells (Supplementary Fig. S5A), indicating that Cdc27 and Tfs1 act in the same GCR pathway. While *cdc27-D2* did not significantly change Rad52 localization at pericentromeric repeats, it did reduce DNA–RNA hybrids at cenR1 (Fig. 5E and F). Strikingly, *rad52-R45K* and *cdc27-D2* synergistically reduced GCR rates to wild-type levels (Fig. 7B), suggesting that Rad52 and Cdc27 collaborate to form and stabilize ADR-loops to cause GCRs.

Cdc27 is a subunit shared by Polδ and Polζ (Fig. 7C and D). Polζ plays a role in translesion synthesis and in microhomology-mediated BIR [85, 86]. The *cdc6-ts2* mutation [87] in a Polδ-specific subunit Cdc6/PolD1 reduced GCR rates in *clr4∆* cells at a semipermissive temperature of 25°C (Fig. 7C). In contrast, loss of Rev1 or Rev3, Polζ-specific subunits, showed no significant effects on GCR rates (Fig. 7D). These results demonstrate that Polδ-dependent BIR, rather than Polζ-dependent BIR, promotes homology-mediated GCRs when heterochromatin is lost and ADR-loops are formed.

## Discussion

Heterochromatin, marked by H3K9me2/3, suppresses GCRs by repressing transcription. However, the mechanism by which transcription causes GCRs remains unclear. In this study, we demonstrated that loss of Clr4 H3K9 methyltransferase in fission yeast results in the accumulation of R-loops at pericentromeric repeats, leading to GCRs. Remarkably, Tfs1, Ubp3, and Seb1, which are involved in transcriptional PBR cycles, were required for the R-loop accumulation and GCRs in *clr4∆* cells. Furthermore, Rad52 and Polδ were required for GCRs. *In vitro*, the Rad52 protein converts R-loops into ADR-loops. Together, our findings suggest that loss of heterochromatin causes transcriptional PBR cycles to accumulate R-loops at pericentromeric repeats. Rad52-dependent SSA between repetitive sequences converts R-loops into ADR-loops, from which Polδ-dependent BIR initiates to copy another side of chromosome arms, resulting in homology-mediated GCRs, such as isochromosome formation (Fig. 7E).

We found that loss of heterochromatin accumulates DNA–RNA hybrids—likely R-loops consisting of DNA–RNA hybrids and ssDNA displaced—at pericentromeric repeats. We provide multiple lines of evidence that loss of heterochromatin causes GCRs by accumulating R-loops at pericentromeric repeats. First, loss of Clr4 or Rik1, but not Swi6, led to the accumulation of R-loops (Fig. 1), consistent with previous findings that loss of Clr4 or Rik1, but not Swi6, increases isochromosome formation [20]. Second, *rad51∆* did not accumulate R-loops (Fig. 1) while it increases isochromosome formation [12, 65]. These results suggest that R-loops are not byproducts of GCRs. Third, overexpression of RNaseH1 in *clr4∆* cells reduced both R-loops and GCRs (Fig. 2). Notably, RNaseH1 overexpression has little effect on R-loops and GCRs in wild-type cells, indicating that R-loops accumulated in *clr4∆* cells are distinct and hypersensitive to RNaseH1. R-loops accumulated in *clr4∆* cells might contain longer ssDNA regions. RPA, which stably binds ssDNA of ≥ 30 nt in length, enhances RNaseH1’s ability to disrupt R-loops, and *clr4∆* increases RPA localization at pericentromeric repeats [88–90]. RNaseH1 preferentially binds R-loops containing RNA with N^6^-methyladenosine (m6A) modification [91]. Thus, it is also possible that R-loops accumulated in *clr4∆* cells carry such a modification. Further research is needed to address these possibilities. Fourth, there is a tight correlation between R-loop and GCR levels. In *clr4∆* cells, *tfs1∆* or *tfs1-DE∆* reduced both cenR1 and cenR2 R-loops, whereas *rnh1-OE, ubp3∆*, or *seb1-1* reduced R-loops at cenR1 but not at cenR2 (Figs. 2, 3, 4, and Supplementary Fig. S4). Accordingly, *tfs1∆* or *tfs1-DE∆* reduced GCRs more significantly than *rnh1-OE, ubp3∆*, or *seb1-1* in *clr4∆* cells. Finally, loss of RNaseH1 and RNaseH2 (i.e. *rnh1∆ rnh201∆*) increased isochromosome formation (Supplementary Fig. S3). These results show that loss of heterochromatin leads to R-loop accumulation at pericentromeric repeats, which in turn causes GCRs.

How does transcription accumulate R-loops at pericentromeric repeats when heterochromatin is lost? Like R-loop accumulation levels, *clr4∆* or *rik1∆* strongly increased RNA transcripts at pericentromeric repeats, but *swi6∆* or *rad51∆* only slightly increased the RNA transcripts (Fig. 1) [92, 93]. Treatment of *clr4∆* cells with a transcription inhibitor, 1,10-phenanthroline, reduced R-loop levels. There seems to be a correlation between transcription and R-loop accumulation levels. However, in *clr4∆* or *rik1∆* cells, R-loop levels were comparable at cenR1 and act1, but transcription levels at cenR1 were only 2% of those at act1, demonstrating that cenR1 transcription tends to form R-loops than act1 transcription. Seb1 causes transcriptional pausing at pericentromeric repeats, and Tfs1 and Ubp3 promote transcriptional restart following pausing and backtracking [22, 25, 26, 28]. In *clr4∆* cells, Tfs1, Ubp3, and Seb1 promote R-loop accumulation specifically at pericentromeric repeats, despite their limited contribution to total transcription levels (Figs. 3 and 4). Therefore, we propose that the transcriptional PBR cycle, but not transcription in general, accumulates genotoxic R-loops at pericentromeric repeats when heterochromatin is lost (Fig. 7E). The PBR cycle may increase the retention time and length of DNA–RNA hybrids, thereby stabilizing R-loops. Supporting this model, R-loops are enriched at promoter-proximal pausing sites and transcription termination sites in mammalian cells [94, 95]. Human Cobra1, a transcriptional pausing factor, also promotes R-loop accumulation at promoter-proximal pausing sites, and TFIIS (the human homolog of Tfs1) localizes to these regions [96, 97]. Contrary to the conventional view that transcriptional restart enhances gene expression, our findings suggest that transcriptional PBR cycles can compromise genome stability by promoting R-loop accumulation at pericentromeric repeats when heterochromatin is lost.

How do R-loops cause homology-mediated GCRs? Non-allelic recombination between pericentromeric inverted repeats can result in GCRs such as isochromosome formation. In yeast, Rad51-dependent HR and Rad52-dependent SSA are the two major pathways of homology-mediated recombination. Genetic analysis demonstrated that Rad52-dependent SSA, but not Rad51-dependent HR, causes GCRs when heterochromatin is lost (Fig. 5). R-loops may recruit Rad52 to pericentromeric repeats because *clr4∆* increased Rad52 localization at cenR1, and RNaseH1 overexpression, *seb1-1*, or *tfs1∆* reduced the Rad52 localization at pericentromeres. Rad52 may localize to pericentromeric repeats during the formation of ADR-loops (Fig. 7E). Indeed, *in vitro* experiments demonstrated that the Rad52 protein, but not the Rad51 protein, promotes ADR-loop formation through the complementary annealing of ssDNA and R-loops (Fig. 6). Further experiments are needed to address whether DNA and RNA sequences and R-loop structures affect Rad52-dependent ADR-loop formation. The *rad52-R45K* mutation, which impairs SSA activity [37], impaired ADR-loop formation (Fig. 6F). *rad52-R45K* also reduced DNA–RNA hybrid levels at cenR1 (Fig. 5F), probably because ADR-loops are more stable than R-loops [98]. Rad52 might also extend the length of DNA–RNA hybrids by DNA–RNA annealing, although it is less efficient than DNA-DNA annealing activity (Supplementary Fig. S7D–G). Together, these data suggest that Rad52-dependent ADR-loop formation at pericentromeric repeats initiates homology-mediated GCRs when heterochromatin is lost.

In theory, both crossover recombination and BIR can form isochromosomes [19]. Crossover recombination is the major pathway for spontaneous isochromosome formation in *rad51∆* cells [65]. However, in *clr4∆* cells, Polδ-dependent BIR rather than crossover recombination is the major pathway to form isochromosomes (Fig. 7 and Supplementary Fig. S9). Polδ may bind ADR-loops, which are produced by Rad52-dependent SSA in the absence of heterochromatin, and initiate DNA synthesis or BIR from the 3′-end of ssDNA within ADR-loops, which stabilized ADR-loops (Fig. 7E). Consistent with this idea, like *rad52-R45K, cdc27-D2* reduced DNA–RNA hybrid levels while it did not reduce Rad52 localization at pericentromeric repeats in *clr4∆* cells (Fig. 5E and F). *rad52-R45K* and *cdc27-D2* synergistically reduced GCR rates in *clr4∆* cells (Fig. 7B), showing genetic interaction between Rad52 and Polδ. These data suggest that homology-mediated GCRs occur through Rad52-dependent ADR-loop formation followed by Polδ-dependent BIR (Fig. 7E). Intriguingly, the structure of ADR-loops resembles that of the replication fork, in that it contains an RNA primer for lagging-strand synthesis and a nascent leading-strand. Therefore, it appears that ADR-loops are the joint molecule competent to support the initiation of Polδ-dependent BIR. When Rad52 produces ADR-loops between homologous sequences at non-allelic rather than allelic positions, subsequent BIR results in homology-mediated GCRs.

Human ICF cells deficient in heterochromatin formation also accumulate R-loops at pericentromeric repeats and exhibit centromere instability [35, 36]. In ICF cells, the XPG endonuclease cleaves R-loops to generate DNA breaks [35]. However, it is unlikely that XPG-dependent DNA breaks are the primary cause of GCRs in fission yeast because the XPG homolog Rad13 was dispensable for GCRs in *clr4∆* cells (Supplementary Fig. S10). In contrast to fission yeast pericentromeres, human pericentromeres consist of numerous copies of short DNA repeats. Thus, the structure of R-loops accumulated in human pericentromeres might differ from that in fission yeast. In human cells, reactive oxygen species induce transcription-coupled HR (TC–HR) through R-loop formation, during which cockayne syndrome protein B (CSB) binds R-loops and recruits Rad52 [99]. However, the CSB homolog Rhp26 was dispensable for GCRs in *clr4∆* cells (Supplementary Fig. S10), suggesting that R-loops created by the PBR cycle at pericentromeric repeats cause GCRs in a manner different from TC-HR. Comparing R-loops across various contexts is crucial to understanding their biological function.

## Supplementary Material

gkaf1455_Supplemental_Files

## Data Availability

The data underlying this article are available in the article and in its online Supplementary data. Uncropped gel images and raw data of DRIP-qPCR, RT-qPCR, ChIP-qPCR, GCR rates, and Rad52 focus analyses have been deposited in the University of Osaka Institutional Knowledge Archive and are accessible at https://doi.org/10.60574/102974. DRIP-seq data have been deposited in the DDBJ/EMBL/GenBank database under accession number PRJDB20605.
