# Supplementary material for: Transcriptional PBR cycles at pericentromeric repeats cause gross chromosomal rearrangements through Rad52-dependent ADR-loop formation
Source: Nucleic Acids Res. 2026 Jan 13;54(1):gkaf1455. doi: 10.1093/nar/gkaf1455 (PMC12795605; doi:10.1093/nar/gkaf1455)
Supplement: gkaf1455_Supplemental_Files [file gkaf1455_supplemental_files.zip › Supplementary Figures.pdf]

Transcriptional PBR cycles at pericentromeric repeats cause gross chromosomal rearrangements through Rad52-dependent ADR-loop formation

Ran Xu, Crystal Tang, Jianfang N. Wang, Daisuke Motooka,
Hideo Tsubouchi, Hiroshi Iwasaki, Takuro Nakagawa

Supplementary Figure S1 to S10

Figure S1

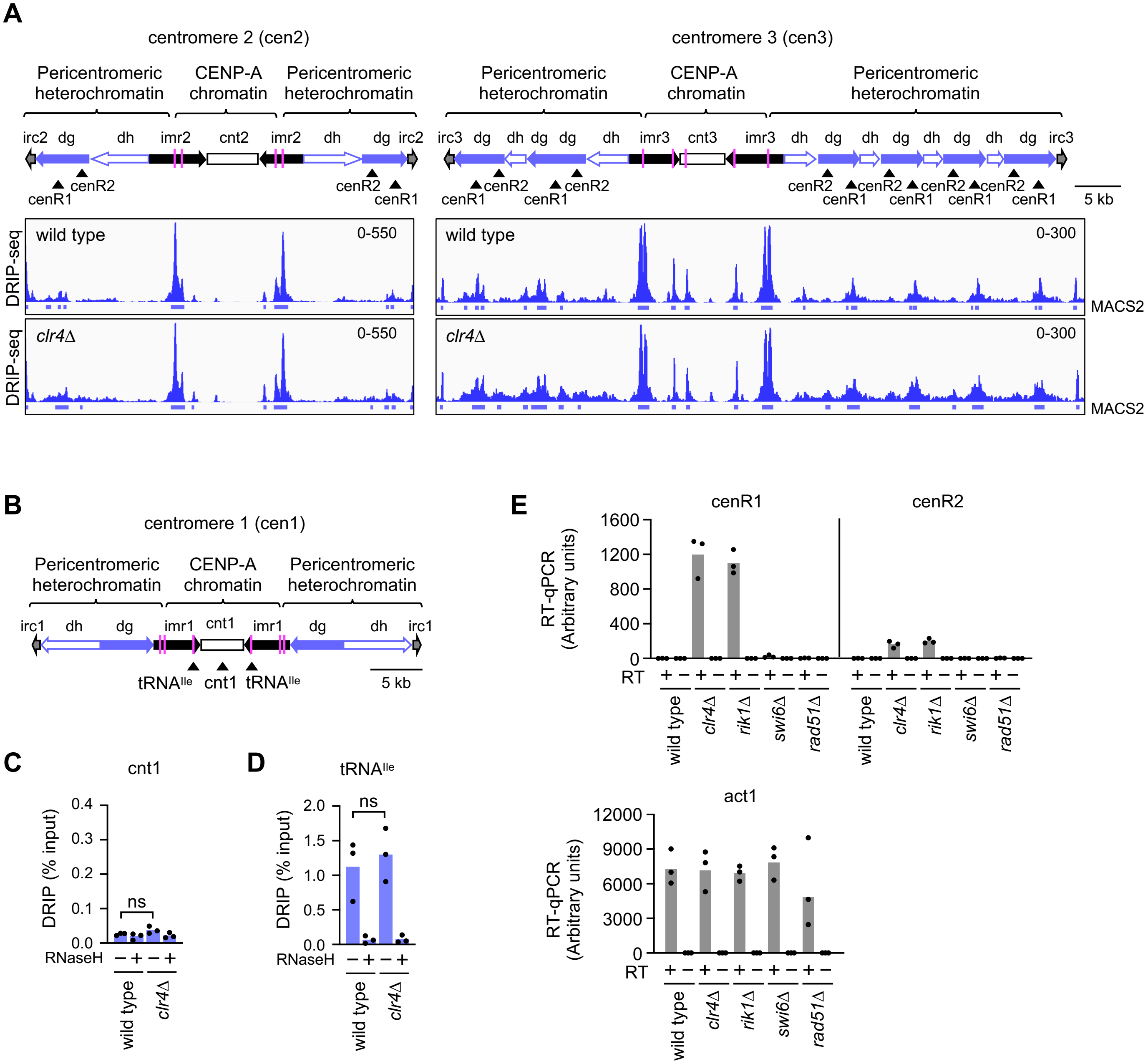

Figure S1. DNA-RNA hybrid and transcription levels.

(**A**) DRIP-seq data of wild-type and *clr4*Δ strains in centromere 2 (cen2) and centromere 3 (cen3). Vertical magenta bars indicate positions of tRNA genes. Arrowheads indicate cenR1 and cenR2 sites amplified in DRIP-qPCR. MACS2 indicates the regions where DNA-RNA hybrids are significantly accumulated. (**B**) Arrowheads indicate DRIP-qPCR amplification sites of cnt1 and $tRNA^{Ile}$ in cen1. DNA-RNA hybrid levels at (**C**) cnt1 and (**D**) $tRNA^{Ile}$ in wild-type and *clr4*Δ strains. Each dot represents a biologically independent experiment (*n* = 3). Bars show the mean. (**E**) RT-qPCR. RNA transcript levels of wild type, *clr4*Δ, *rik1*Δ, *swi6*Δ, and *rad51*Δ cells. RNA levels were expressed as arbitrary units based on the qPCR standard curve. RT, reverse transcription. Each dot represents a biologically independent experiment (*n* = 3). Bars show the mean.

Figure S2

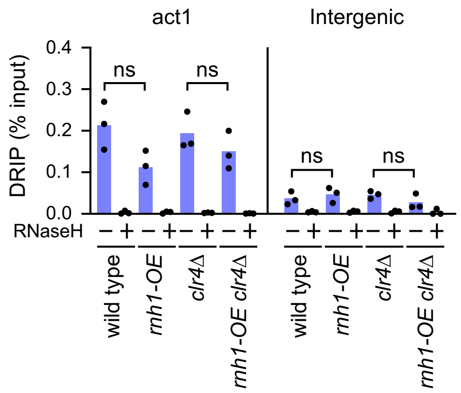

Figure S2. DNA-RNA hybrid levels at act1 and Intergenic sites.

DNA-RNA hybrid levels at act1 and Intergenic sites in wild-type, *rnh1-OE*, *clr4*Δ, and *rnh1-OE clr4*Δ strains. Each dot represents a biologically independent experiment (*n* = 3). Bars show the mean.

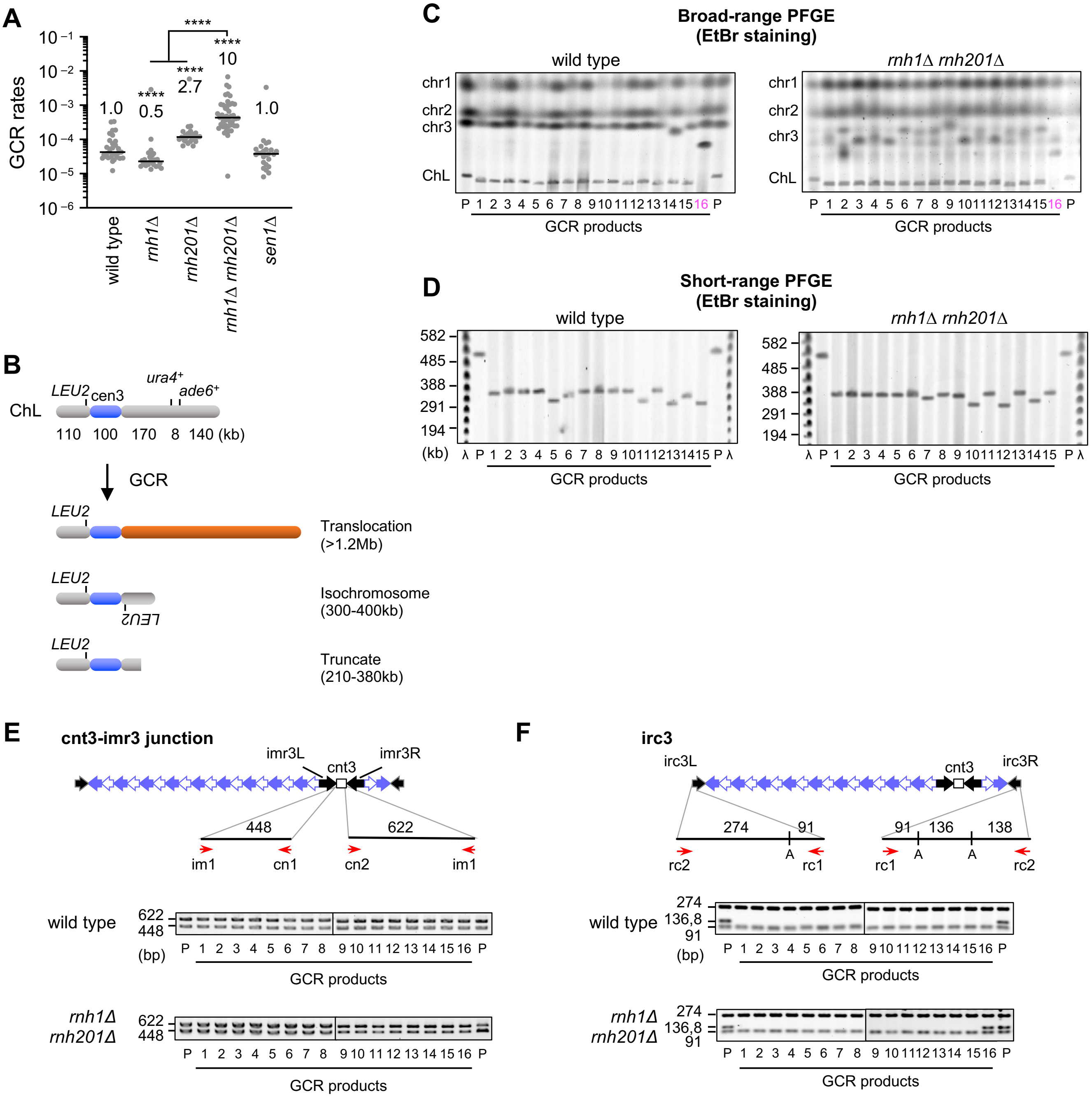

Figure S3. Rnh1 and Rnh201 suppress isochromosome formation at centromeres.
(**A**) GCR rates of wild-type, *rnh1*Δ, *rnh201*Δ, *rnh1*Δ *rnh201*Δ, and *sen1*Δ strains. Each dot represents a biologically independent experiment. Lines show the median. GCR rates relative to wild type are shown at the top of each column. (**B**) Three types of Leu$^+$ Ura$^-$ Ade$^-$ GCR products are illustrated. (**C**-**D**) Chromosomal DNAs prepared from the parental and GCR clones of wild-type and *rnh1*Δ *rnh201*Δ strains were separated by (**C**) Broad-range PFGE and (**D**) Short-range PFGE and stained with EtBr. (**C**) Positions of chr1, chr2, chr3, and ChL are indicated on the left of each gel image. Translocations are shown in magenta. P, Parental. (**D**) Sizes of the λ phage DNA ladder are shown on the left of each image. (**E**-**F**) PCR analysis of GCR products recovered from the agarose gel to determine whether the breakpoint is present in centromere repeats. Red arrows indicate the position of the primers to amplify (**E**) cnt3-imr3 junctions and (**F**) irc3. irc3 PCR products were digested with the ApoI restriction enzyme before standard agarose gel electrophoresis. A, ApoI.

Figure S4

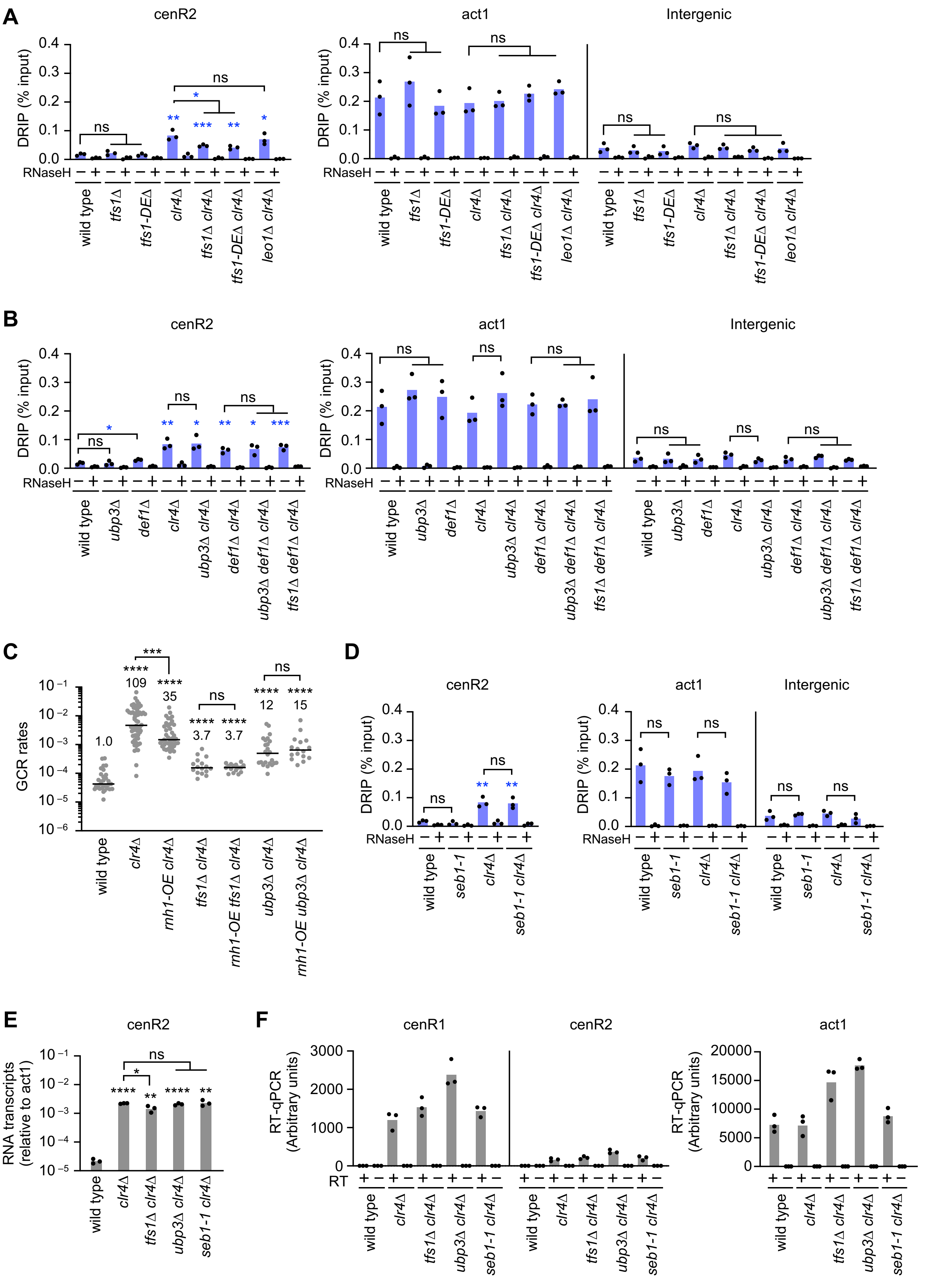
A
cenR2
act1
Intergenic
DRIP (% input)
0.4
0.3
0.2
0.1
0.0
RNaseH
ns
*
**
***
wild type
tfs1Δ
tfs1-DEΔ
clr4Δ
tfs1Δ clr4Δ
tfs1-DEΔ clr4Δ
leo1Δ clr4Δ
B
cenR2
act1
Intergenic
DRIP (% input)
RNaseH
wild type
ubp3Δ
def1Δ
clr4Δ
ubp3Δ clr4Δ
def1Δ clr4Δ
ubp3Δ def1Δ clr4Δ
tfs1Δ def1Δ clr4Δ
C
GCR rates
1.0
109
35
3.7
3.7
12
15
****
***
ns
wild type
clr4Δ
rnh1-OE clr4Δ
tfs1Δ clr4Δ
rnh1-OE tfs1Δ clr4Δ
ubp3Δ clr4Δ
rnh1-OE ubp3Δ clr4Δ
D
cenR2
act1
Intergenic
DRIP (% input)
RNaseH
wild type
seb1-1
clr4Δ
seb1-1 clr4Δ
E
cenR2
RNA transcripts (relative to act1)
wild type
clr4Δ
tfs1Δ clr4Δ
ubp3Δ clr4Δ
seb1-1 clr4Δ
F
cenR1
cenR2
act1
RT-qPCR (Arbitrary units)
3000
2000
1000
0
20000
15000
10000
5000
RT
wild type
clr4Δ
tfs1Δ clr4Δ
ubp3Δ clr4Δ
seb1-1 clr4Δ

Figure S4

Figure S4. DNA-RNA hybrid, GCR, and transcription levels in *tfs1*, *ubp3*, and *seb1* mutants.
(**A**) DNA-RNA hybrid levels at cenR2, act1, and Intergenic sites in wild-type, *tfs1*Δ, *tfs1-DE*Δ, *clr4*Δ, *tfs1*Δ *clr4*Δ, *tfs1-DE*Δ *clr4*Δ, and *leo1*Δ *clr4*Δ strains. (**B**) DNA-RNA hybrid levels in wild-type, *ubp3*Δ, *def1*Δ, *clr4*Δ, *ubp3*Δ *clr4*Δ, *def1*Δ *clr4*Δ, *ubp3*Δ *def1*Δ *clr4*Δ, and *tfs1*Δ *def1*Δ *clr4*Δ strains. Each dot represents a biologically independent experiment (*n* = 3). Bars show the mean. (**C**) GCR rates of wild-type, *clr4*Δ, *rnh1-OE clr4*Δ, *tfs1*Δ *clr4*Δ, *rnh1-OE tfs1*Δ *clr4*Δ, *ubp3*Δ *clr4*Δ, and *rnh1-OE ubp3*Δ *clr4*Δ strains. Each dot represents a biologically independent experiment. Lines show the median. GCR rates relative to wild type are shown at the top of each column. (**D**) DNA-RNA hybrid levels in wild-type, *seb1-1*, *clr4*Δ, and *seb1-1 clr4*Δ strains. (**E**) RT-qPCR. RNA transcript levels of cenR2 relative to act1 are shown in wild-type, *clr4*Δ, *tfs1*Δ *clr4*Δ, *ubp3*Δ *clr4*Δ, and *seb1-1 clr4*Δ strains. Each dot represents a biologically independent experiment (*n* = 3). Bars show the mean. (**F**) RT-qPCR. RNA transcript levels of wild-type, *clr4*Δ, *tfs1*Δ *clr4*Δ, *ubp3*Δ *clr4*Δ, and *seb1-1 clr4*Δ cells. RNA levels were expressed as arbitrary units based on the qPCR standard curve.

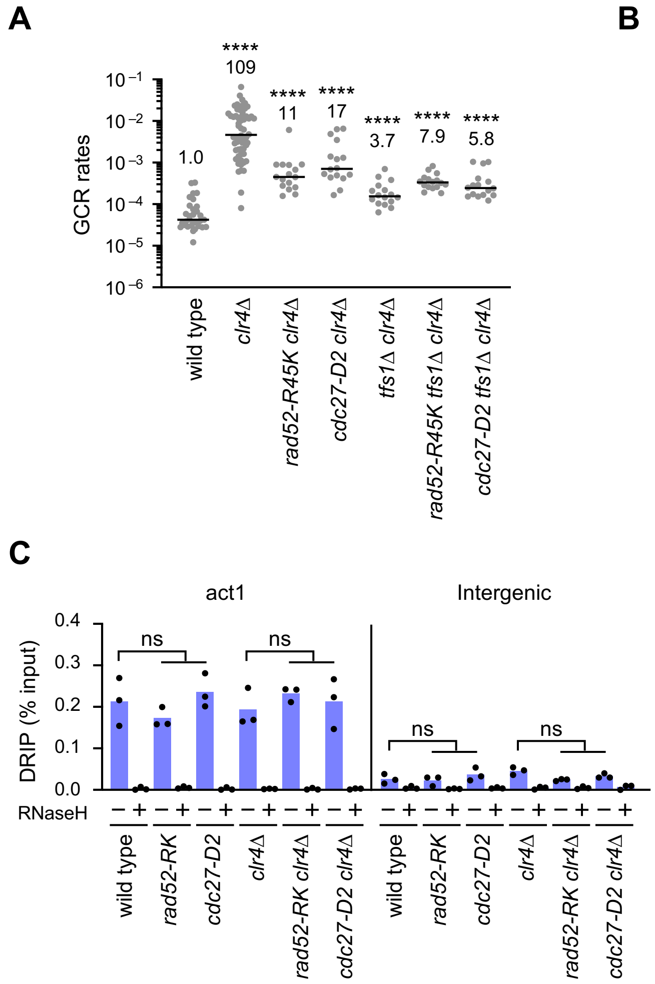

Figure S5. Rad52 and Cdc27 promote GCRs in *clr4*Δ cells.

(**A**) GCR rates of wild-type, *clr4*Δ, *rad52-R45K clr4*Δ, *cdc27-D2 clr4*Δ, *tfs1*Δ *clr4*Δ, *rad52-R45K tfs1*Δ *clr4*Δ, and *cdc27-D2 tfs1*Δ *clr4*Δ strains. Each dot represents a biologically independent experiment. Lines show the median. GCR rates relative to wild type are shown at the top of each column. (**B**) Rad52-6His3Flag localization at the act1 site in no-tag strain and *rad52-6His3Flag* strains of wild-type, *clr4*Δ, *rnh1-OE clr4*Δ, *seb1-1 clr4*Δ*, tfs1*Δ *clr4*Δ, and *cdc27-D2 clr4*Δ. The recovery relative to the no-tag control is shown. Each dot represents an independent experiment (*n* = 3). Bars show the mean. (**C**) DNA-RNA hybrid levels at act1 and Intergenic sites in wild-type, *rad52-R45K*, *cdc27-D2*, *clr4*Δ, *rad52-R45K clr4*Δ, and *cdc27-D2 clr4*Δ strains. Each dot represents a biologically independent experiment (*n* = 3). Bars show the mean.

Figure S6

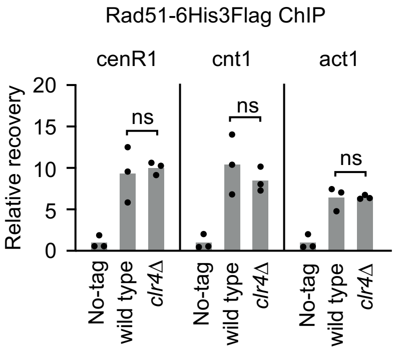

Figure S6. Rad51 ChIP.
Rad51 ChIP experiments using no-tag strain and *rad51-6His3Flag* strains of wild-type and *clr4*Δ. The recovery relative to the no-tag control is shown. Each dot represents a biologically independent experiment (n = 3). Bars show the mean.

Figure S7

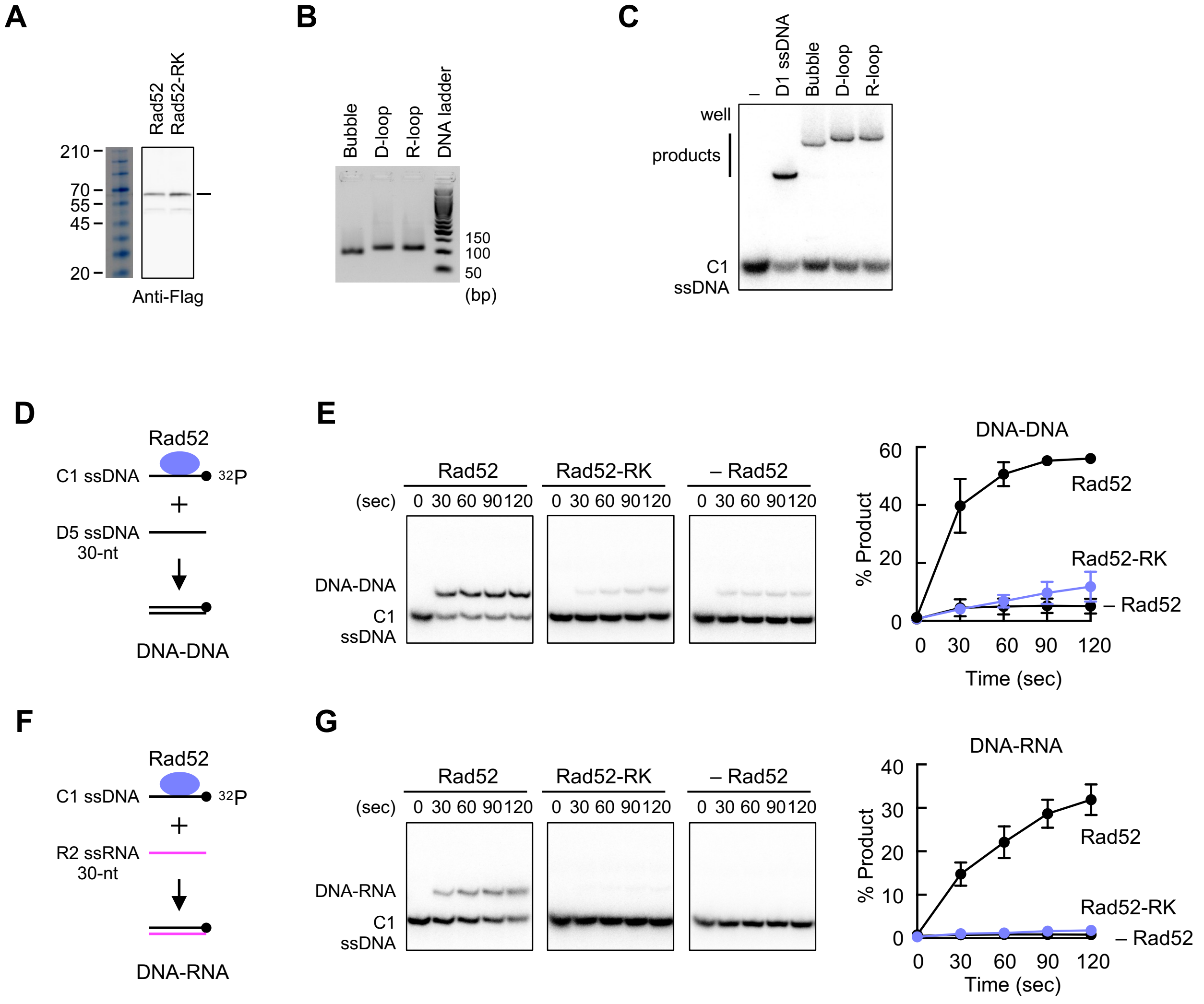

Figure S7. Rad52-dependent annealing assays.

(**A**) Purified wild-type Rad52 and mutant Rad52-R45K proteins were separated by 10% SDS-PAGE and detected by a Western blot using anti-Flag antibodies. Sizes of CLEARLY Stained Protein Ladder (Takara) are indicated on the left of the panel. (**B**) Bubble, D-loop, and R-loop were separated by 3% agarose gel (Nacalai Tesque, 01153-22) in 1 × TBE buffer and stained with EtBr. Sizes of the 50-bp DNA ladder are indicated on the right of the panel. (**C**) Rad52 promotes annealing between C1 and D1 ssDNA, Bubble, D-loop, or R-loop. The annealing products of Bubble showed higher mobility than R-loop and D-loop. The reaction mixture contains 0.3 nM of C1, 0.3 nM of the substrate, and 1.35 nM of Rad52. (**D**) Rad52-dependent DNA-DNA annealing. Rad52 was pre-incubated with C1 labeled with ^{32}P at the 5'-end. The reaction was initiated by adding D5 ssDNA. The reaction mixture contains 0.3 nM of C1, 0.3 nM of D5 ssDNA molecules, and 1.35 nM of Rad52. The reaction product was applied to 12% non-denaturing PAGE in 1 × TBE buffer. Radiation signals were detected using a phosphorimager FLA7000. (**E**) The gel image. Percentages of annealing products over time are shown in the graph. Mean ± SD of 3 independent experiments. (**F**) Rad52-dependent DNA-RNA annealing. The reaction mixture contains 0.3 nM of C1, 0.3 nM of R2 ssRNA molecules, and 1.35 nM of Rad52. (**G**) The gel image. Percentages of annealing products over time are shown in the graph.

Figure S8

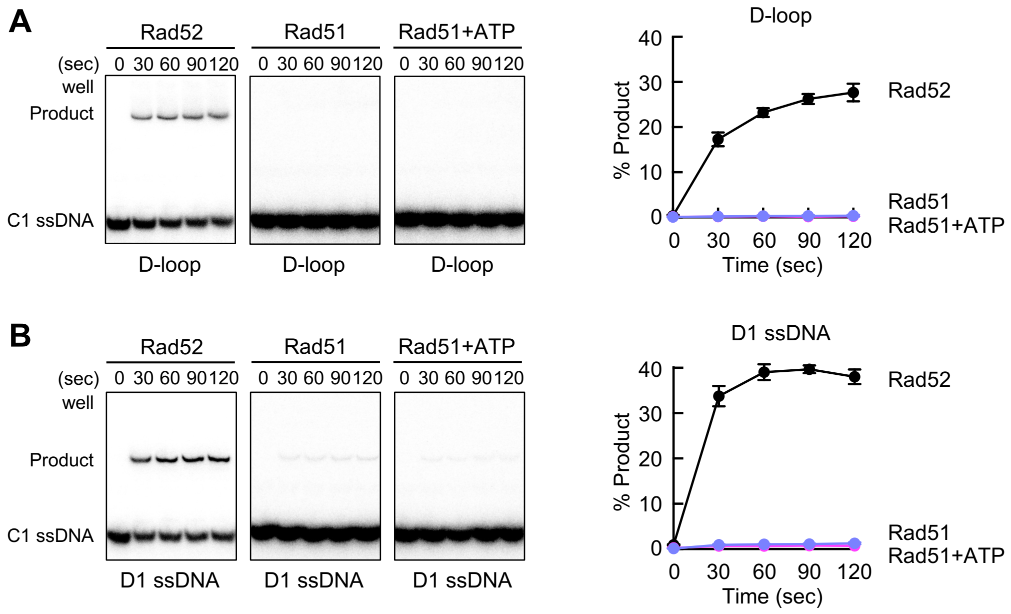

Figure S8. Comparison between Rad52 and Rad51 in DNA annealing.

(**A**) DNA annealing between C1 ssDNA and D-loop in the presence of 1.35 nM Rad52, 4.5 nM Rad51, or 4.5 nM Rad51 and 1 mM ATP. Percentages of annealing products over time are shown in the graphs. Mean ± SD of 3 independent experiments.

(**B**) DNA annealing between C1 and D1 ssDNAs.

Figure S9

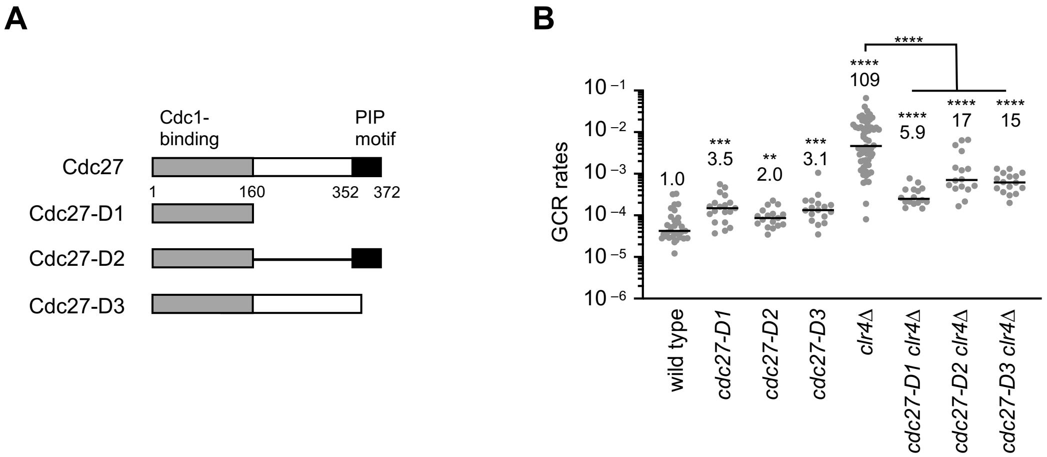

Figure S9. Cdc27 truncations reduced GCR rates in *clr4*Δ cells.
(**A**) The Cdc27-D1, -D2, and -D3 truncates. (**B**) GCR rates of wild-type, *cdc27-D1*, *cdc27-D2*, *cdc27-D3*, *clr4*Δ, *cdc27-D1 clr4*Δ, *cdc27-D2 clr4*Δ, and *cdc27-D3 clr4*Δ strains. Each dot represents a biologically independent experiment. Lines show the median. GCR rates relative to wild type are shown at the top of each column.

Figure S10

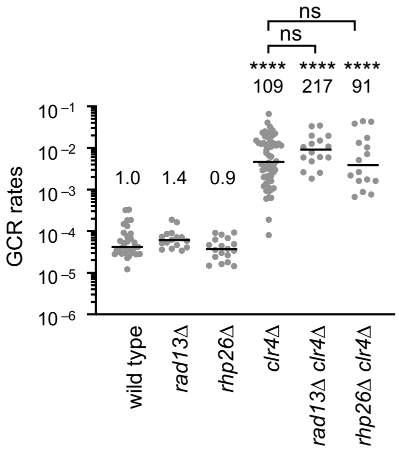

Figure S10. Rad13 and Rhp26 are not essential for GCRs in *clr4Δ* cells.

GCR rates of wild-type, *rad13Δ, rhp26Δ, clr4Δ*, *rad13Δ clr4Δ,* and *rhp26Δ clr4Δ* strains. Each dot represents a biologically independent experiment. Lines show the median. GCR rates relative to wild type are shown at the top of each column.
